# Supplementary figures and images for: Regulatory network-based imputation of dropouts in single-cell RNA sequencing data
Source: PLoS Comput Biol. 2022 Feb 17;18(2):e1009849. doi: 10.1371/journal.pcbi.1009849 (PMC8890719; doi:10.1371/journal.pcbi.1009849)

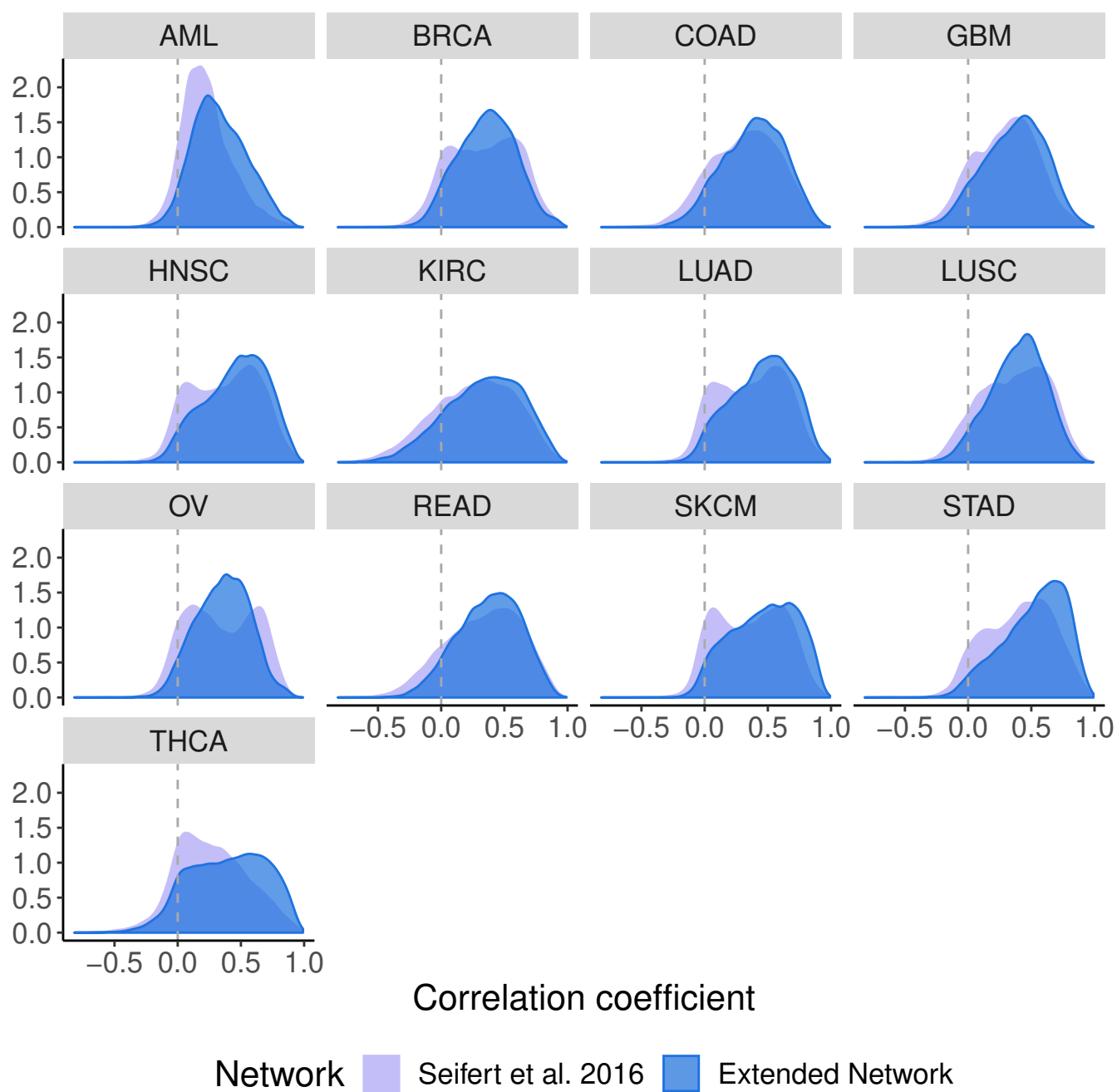

Supplement: S1 Fig — For each gene its expression was predicted in a given tumor sample using the measured expression values of all detected predictors in the model. Subsequently, observed and predicted values were correlated across all samples from one cohort. The plots show the distributions of Pearson’s correlation scores across all genes common between the network model and the respective TCGA dataset. Although there is variation with respect to how well genes in different tumor entities can be predicted, the distributions are always strongly skewed in favour of positive correlations. This trend is enhanced with the new model presented here. AML—Acute Myeloid Leukemia; BRCA—Breast Invasive Carcinoma; COAD—Colon Adenocarcinoma; GBM—Glioblastoma Multiforme; HNSC—Head and Neck Squamous Cell Carcinoma; KIRC—Kidney Renal Clear Cell Carcinoma; LUAD—Lung Adenocarcinoma; LUSC—Lung Squamous Cell Carcinoma; OV—Ovarian Serous Cystadenocarcinoma; READ—Rectum Adenocarcinoma; SKCM—Skin Cutaneous Melanoma; STAD—Stomach Adenocarcinoma; THCA—Thyroid Carcinoma. (PDF) [file pcbi.1009849.s005.pdf]

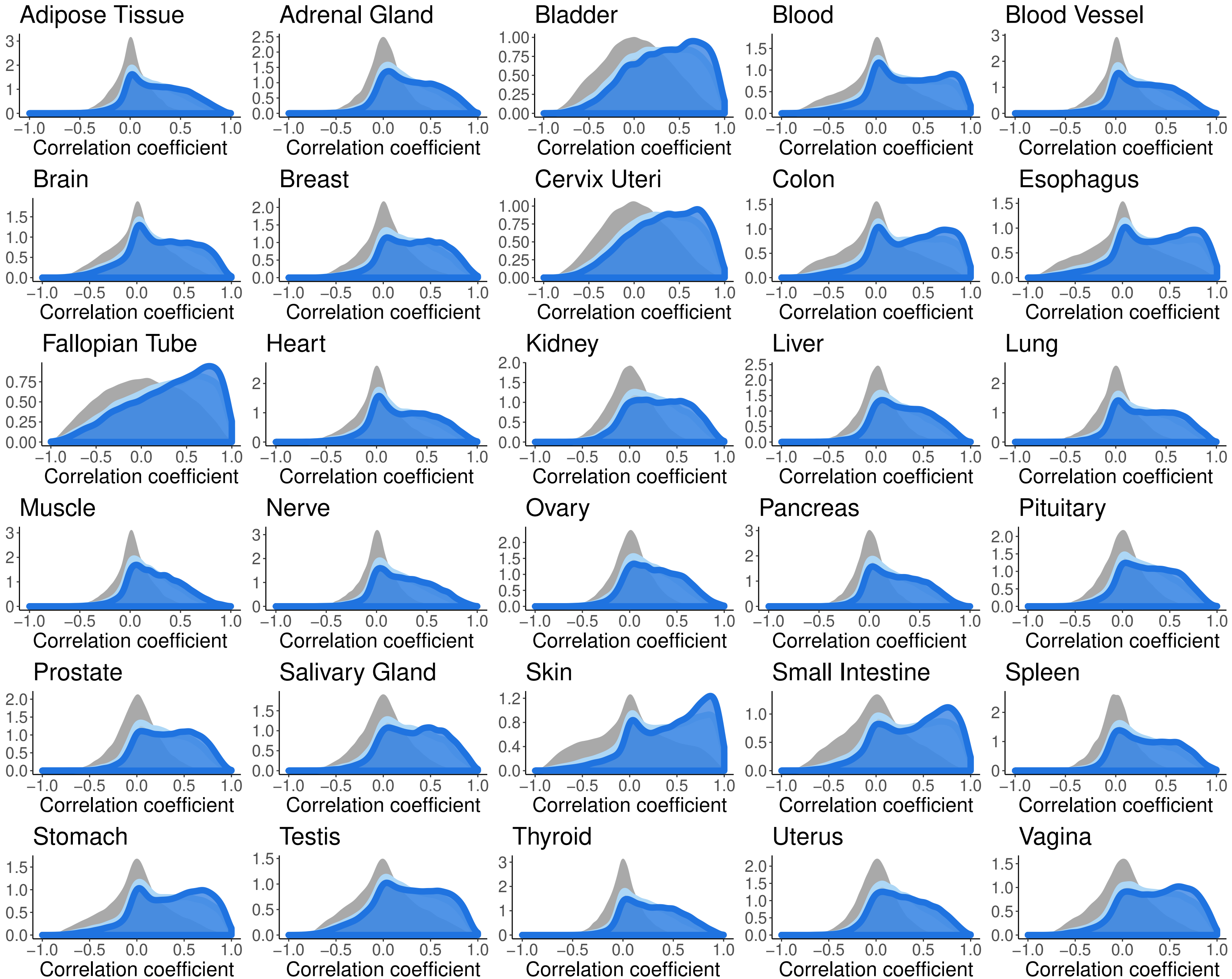

Supplement: S2 Fig — For each gene its expression was predicted in a given healthy tissue sample using the measured expression values of all detected predictors in the model. Subsequently, observed and predicted values were correlated across all samples from one tissue. The plots show the distributions of Pearson’s correlation scores across all genes common between the network model and the GTEx data. (PDF) [file pcbi.1009849.s006.pdf]

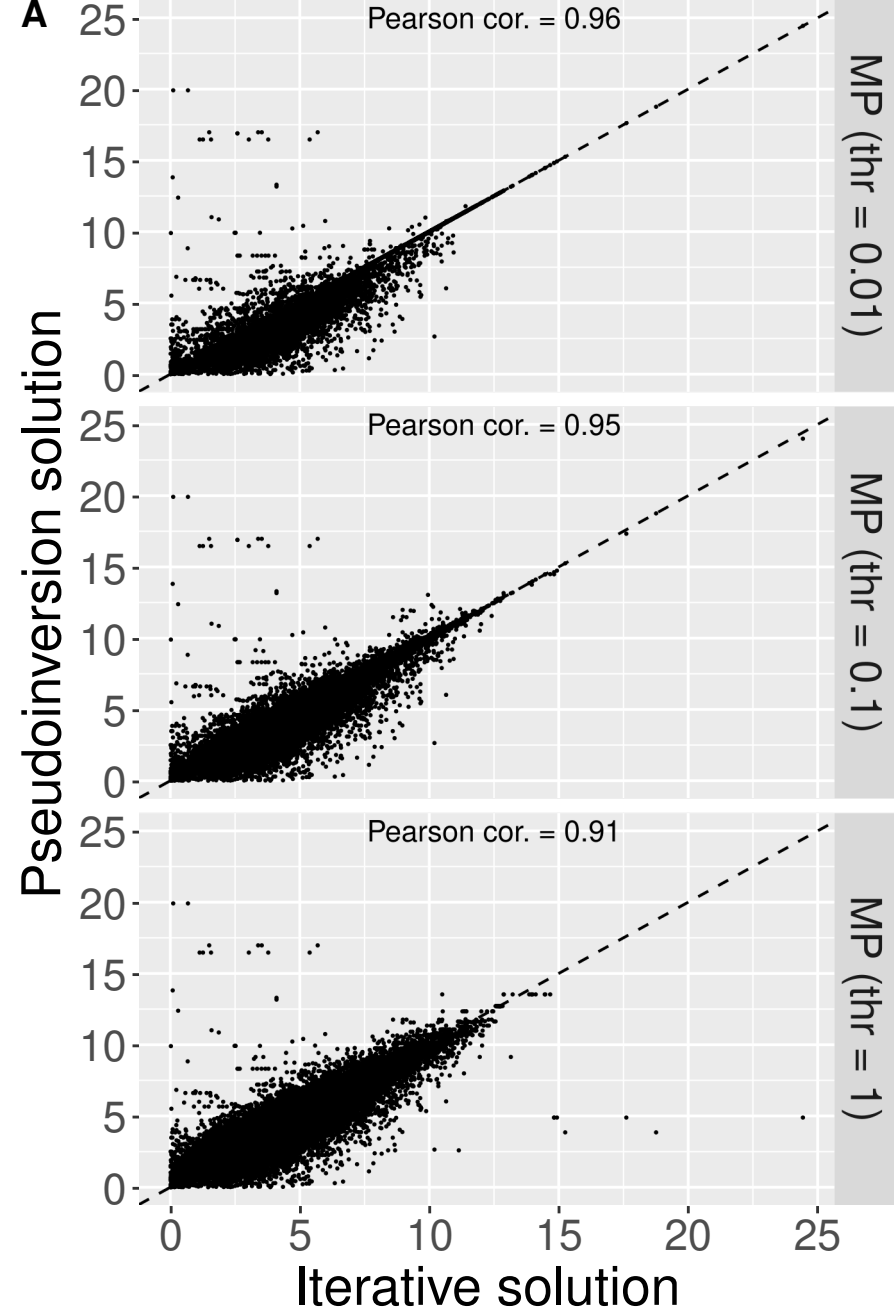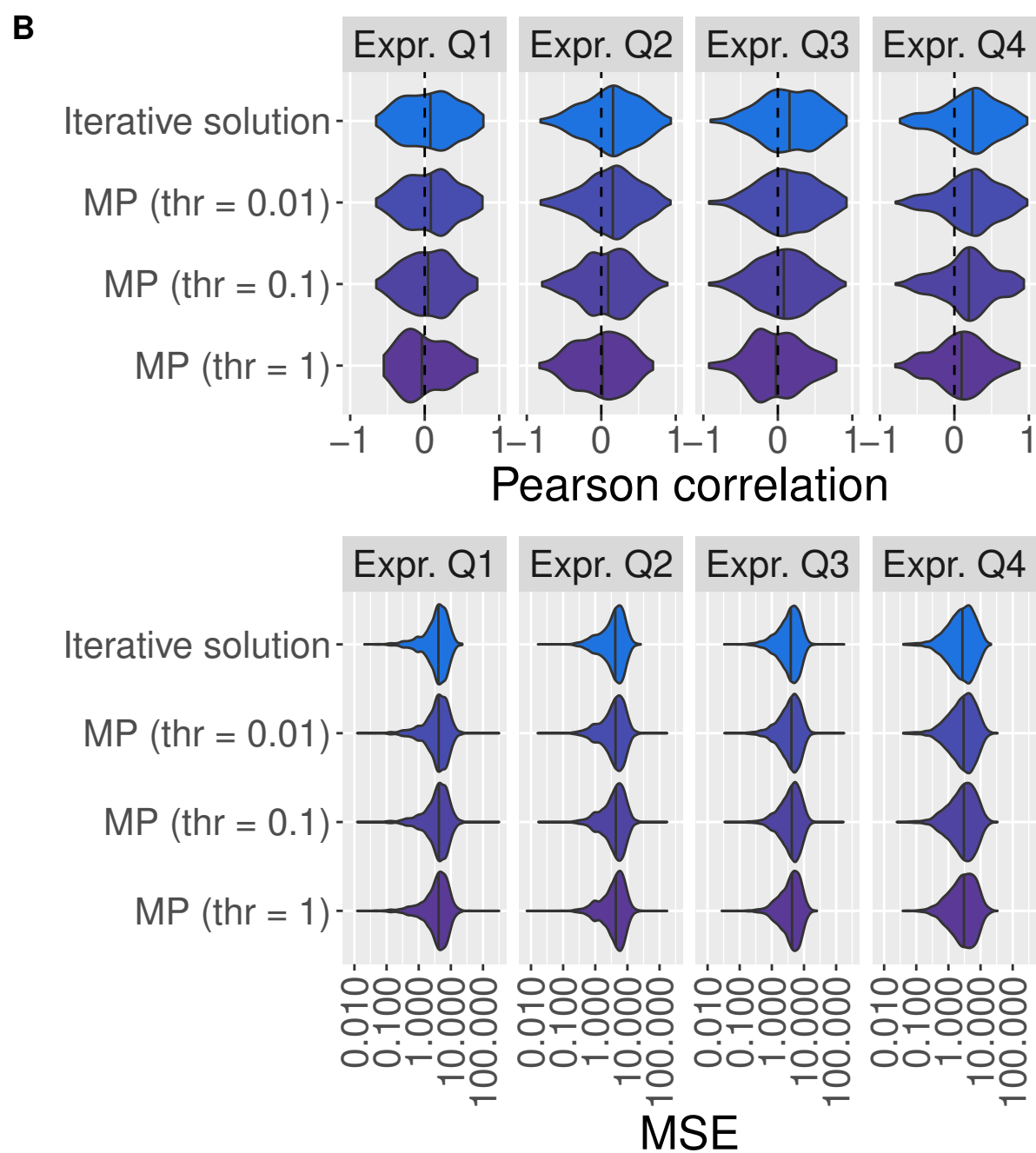

Supplement: S3 Fig — A) Correlation between the results of the iterative approach (x axis) and the Moore-Penrose pseudoinversion (y-axis), across the 20 random cells. B) Imputation performance per gene using the iterative approach and MP with different tolerance thresholds (Pearson correlation, top, and MSE, bottom), separated by expression quartile on the masked data. The higher the tolerance threshold, the fewer singular values are used for the pseudoinversion. Results were limited to imputations performed by all methods. (PDF) [file pcbi.1009849.s007.pdf]

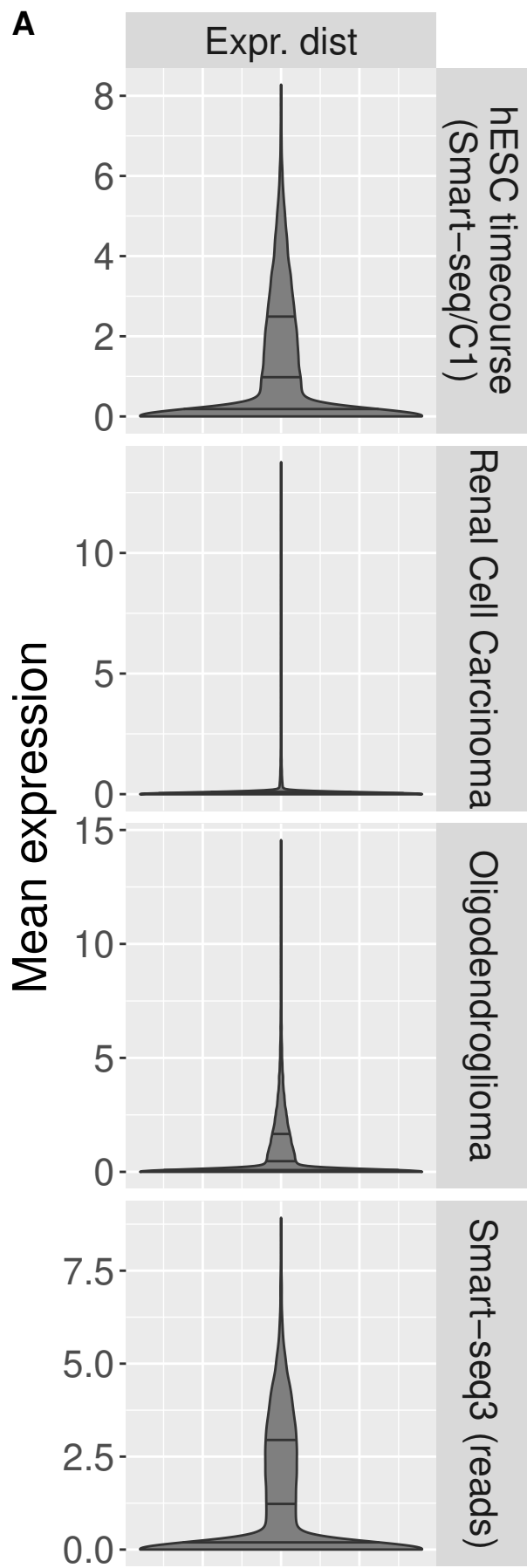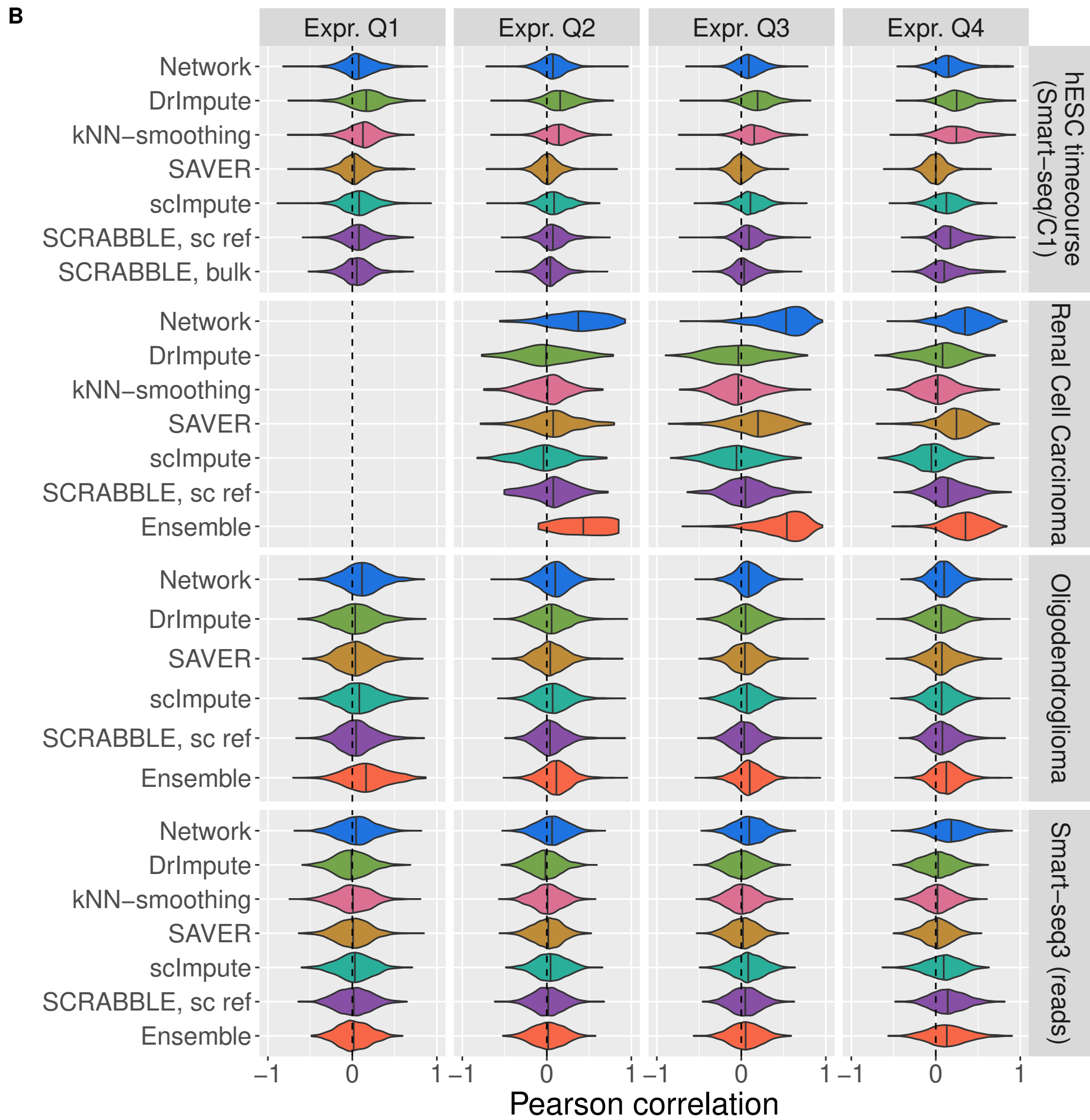

Supplement: S4 Fig — A) Distribution of average expression levels in each dataset. Quartiles are represented by vertical lines. B) Pearson correlation coefficient, for each gene, between the imputation by the specified method and the original values before masking. Only values that could be imputed by all methods were used for correlation computation. Expression quartiles are determined for each dataset separately, on the masked data. (PDF) [file pcbi.1009849.s008.pdf]

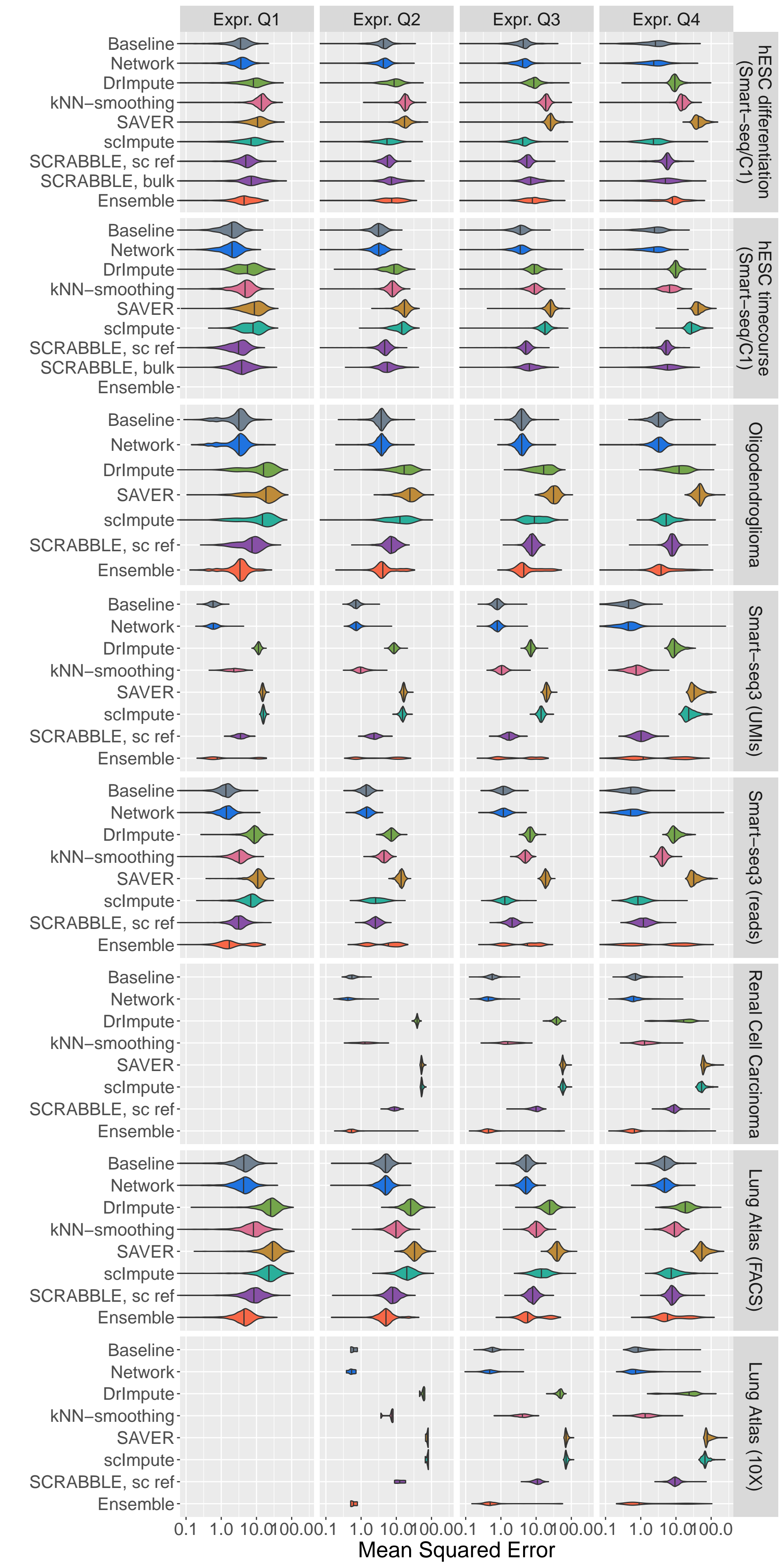

Supplement: S5 Fig — Only values that could be imputed by all methods were used for MSE computation. Expression quartiles are determined for each dataset separately, on the masked data. The x axis is presented log-transformed and was cropped at 0.1 to exclude the low-MSE tail from visualization and facilitate result comparison. (PDF) [file pcbi.1009849.s009.pdf]

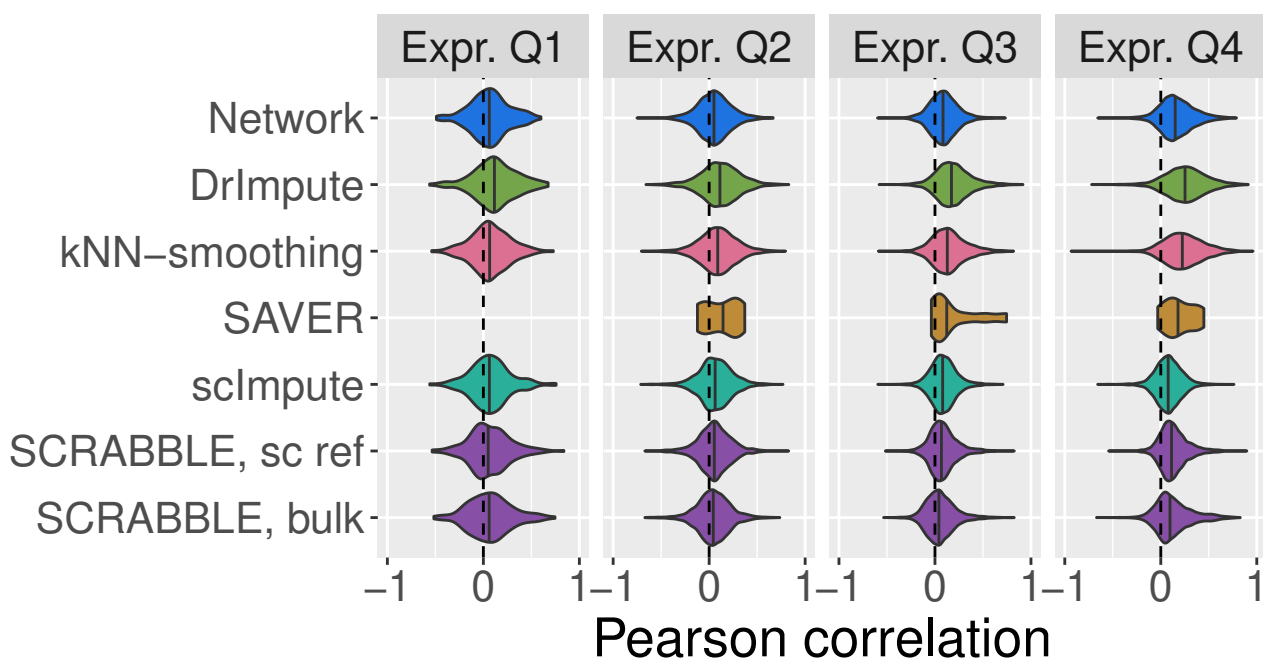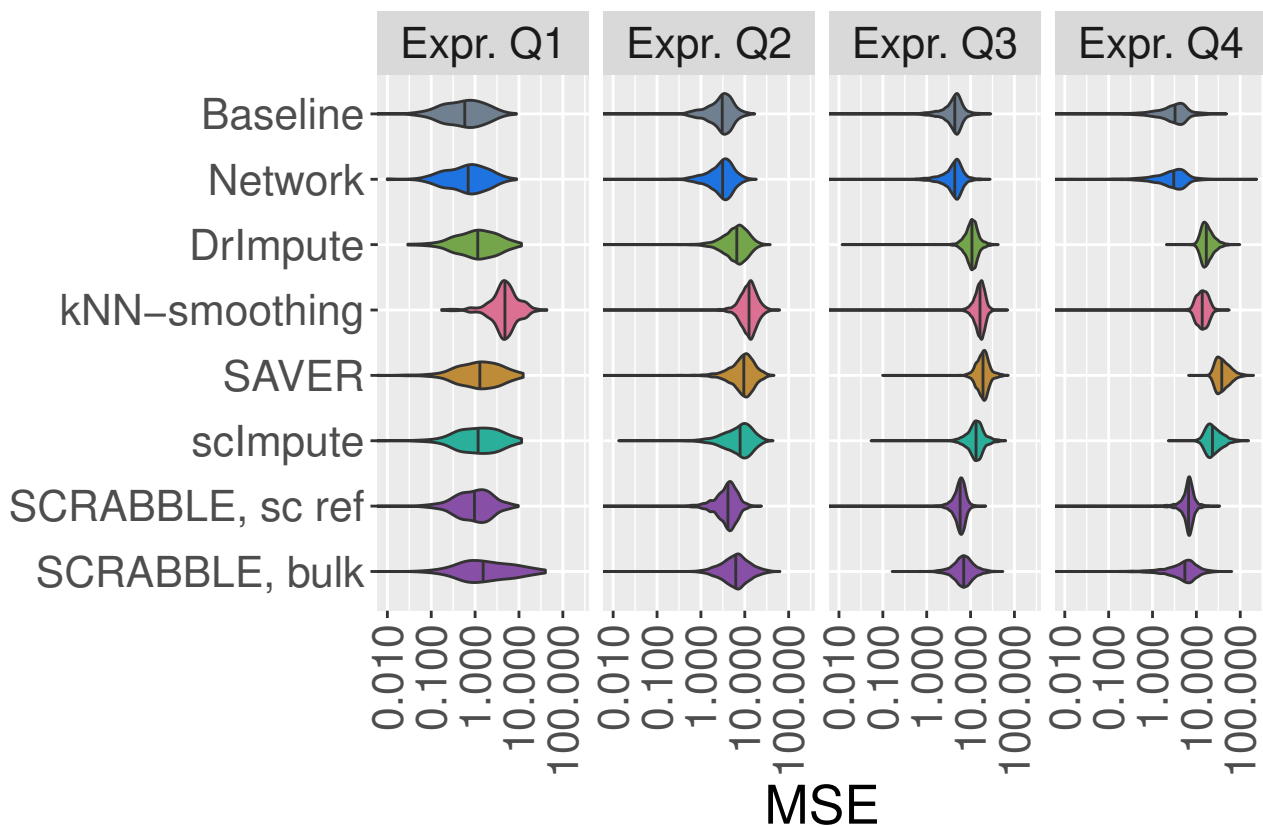

Supplement: S6 Fig — Correlation coefficient between imputed and original values (top) and MSE of imputation (bottom), upon random masking of 30% of the quantified genes in each cell in the hESC differentiation dataset. The Baseline method computes the average expression of a gene across all cells and it is not using the information of any other genes. Hence, one would assume that its error should be independent of the number of missing genes per cell. This is however not the case due to a biased gene sampling: cells with few detected genes will preferentially report values for highly expressed genes, whereas cells with many detected genes will represent a less biased sample of the whole transcriptome. This is affecting the performance, which thus is slightly dependent on the number of missing genes per cell. Values were restricted to imputations performed by all tested methods. (PDF) [file pcbi.1009849.s010.pdf]

Pearson correlation

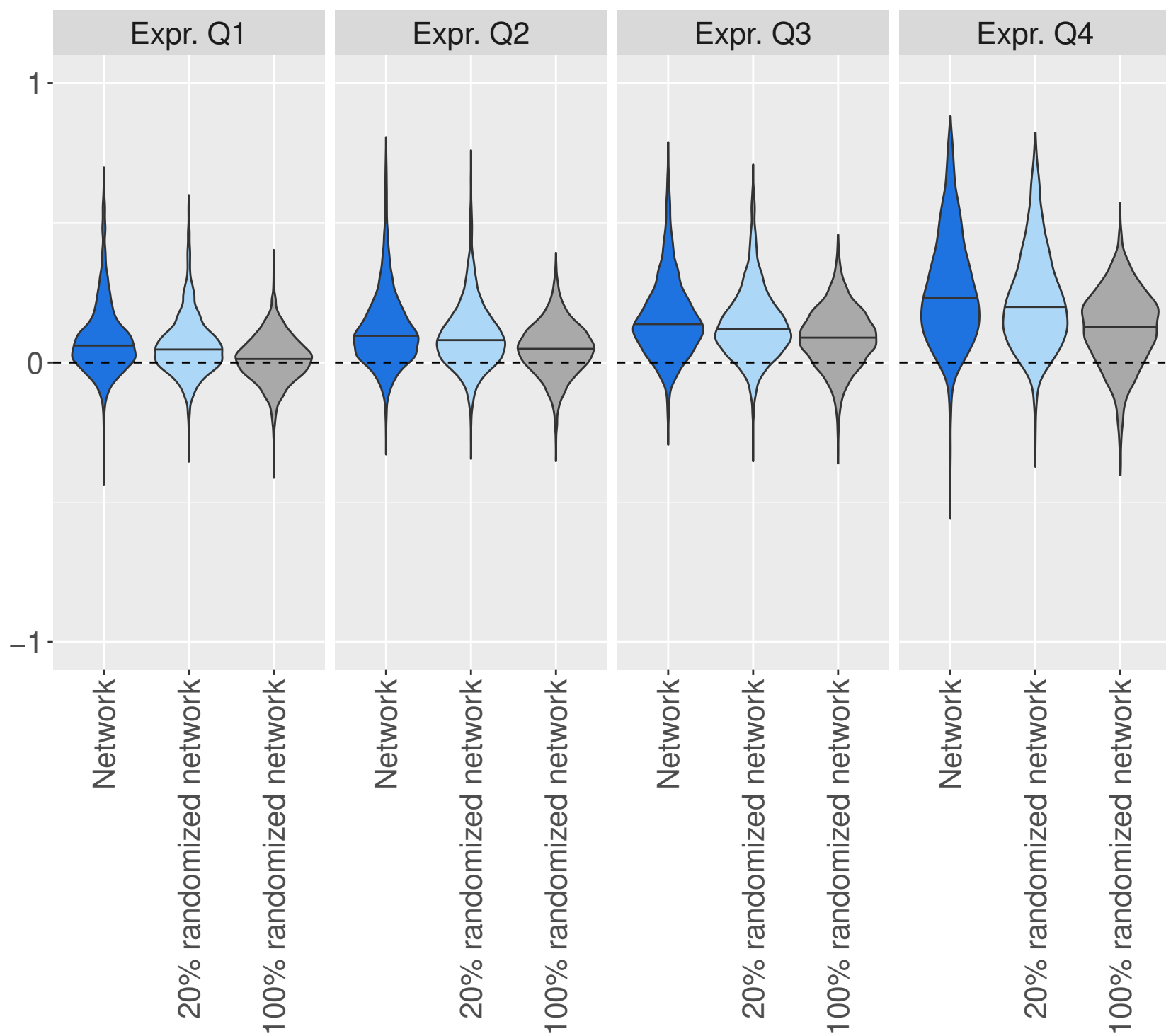

Supplement: S7 Fig — Dropout imputation was performed using the network described here (blue), a partially randomized network (light blue) and a fully randomized network (grey). (PDF) [file pcbi.1009849.s011.pdf]

non-UMI (hESC differentiation)

**A)**

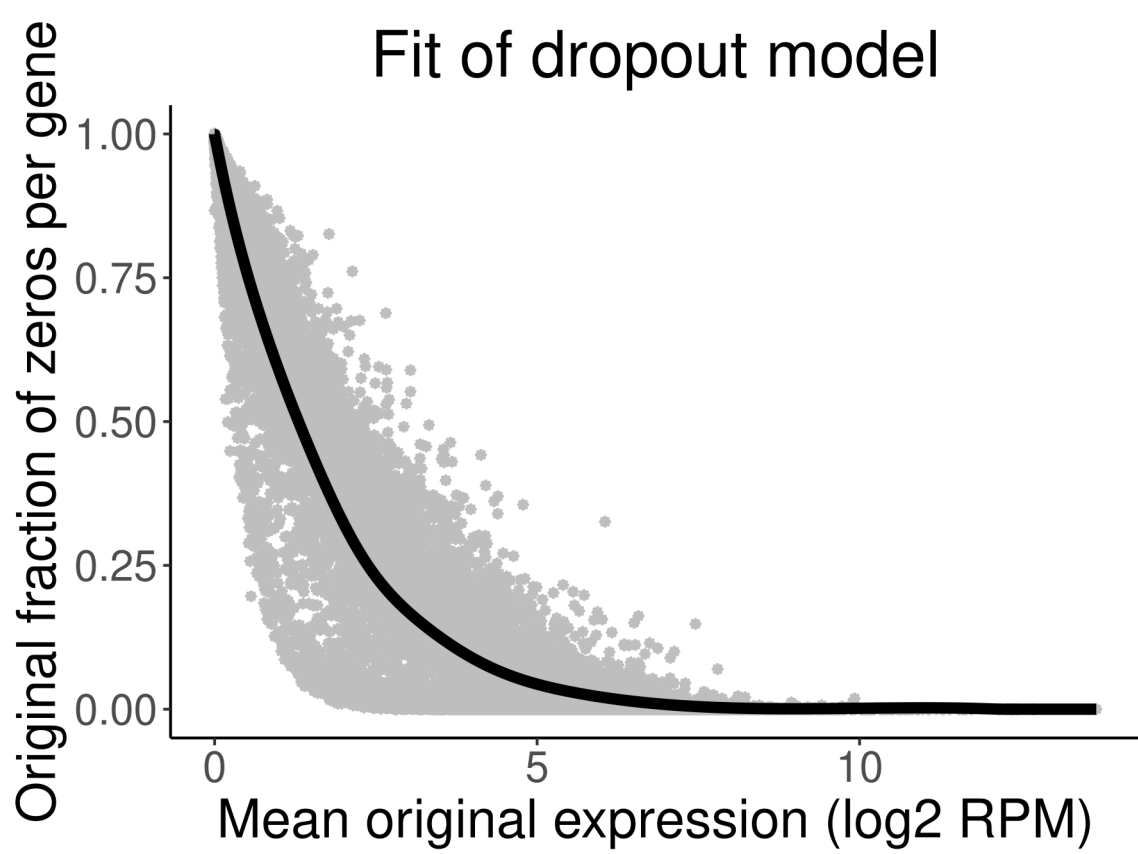

**C)**

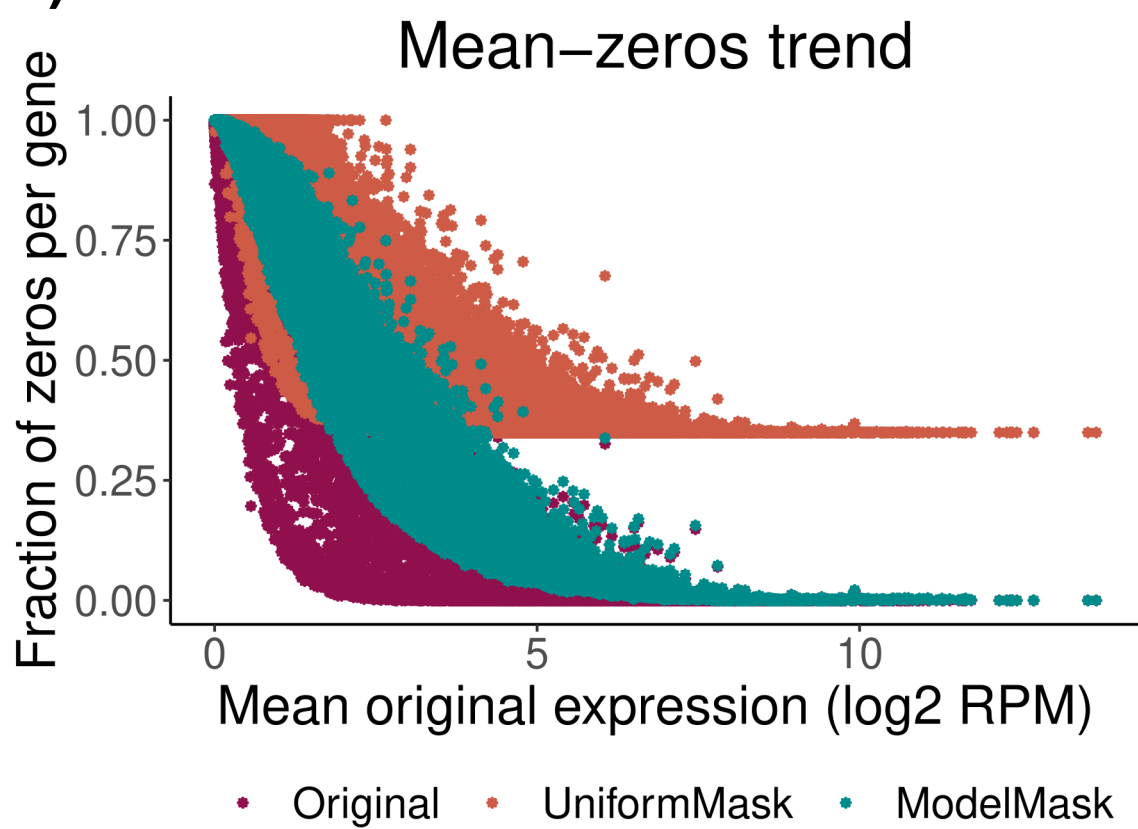

**E)**

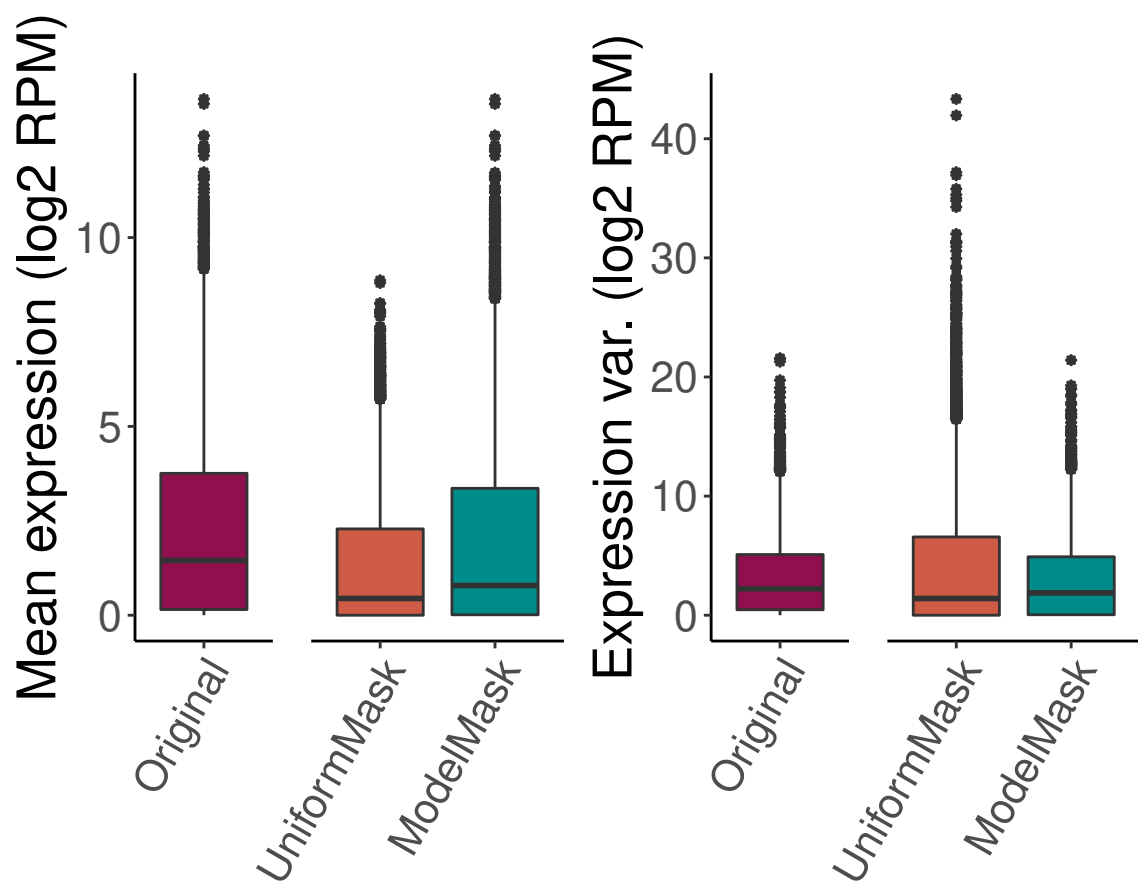

**G)**

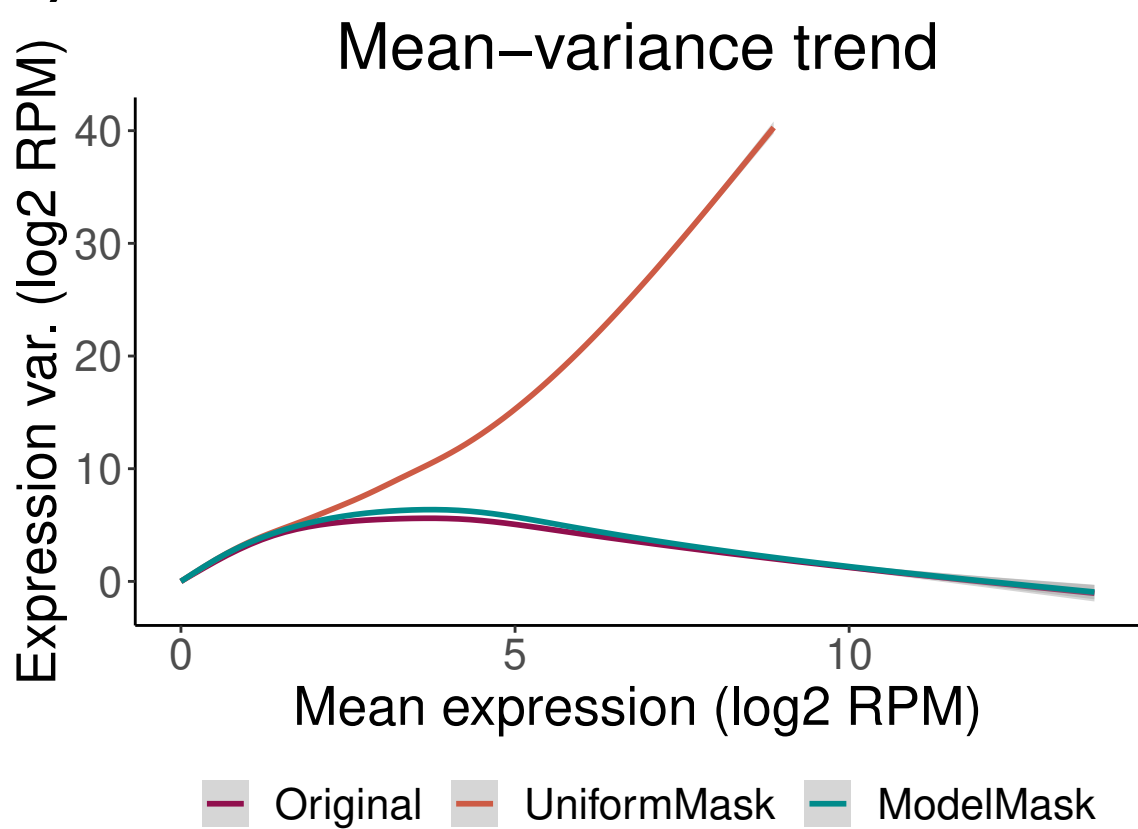

UMI (Healthy pancreas indiv. 1)

**B)**

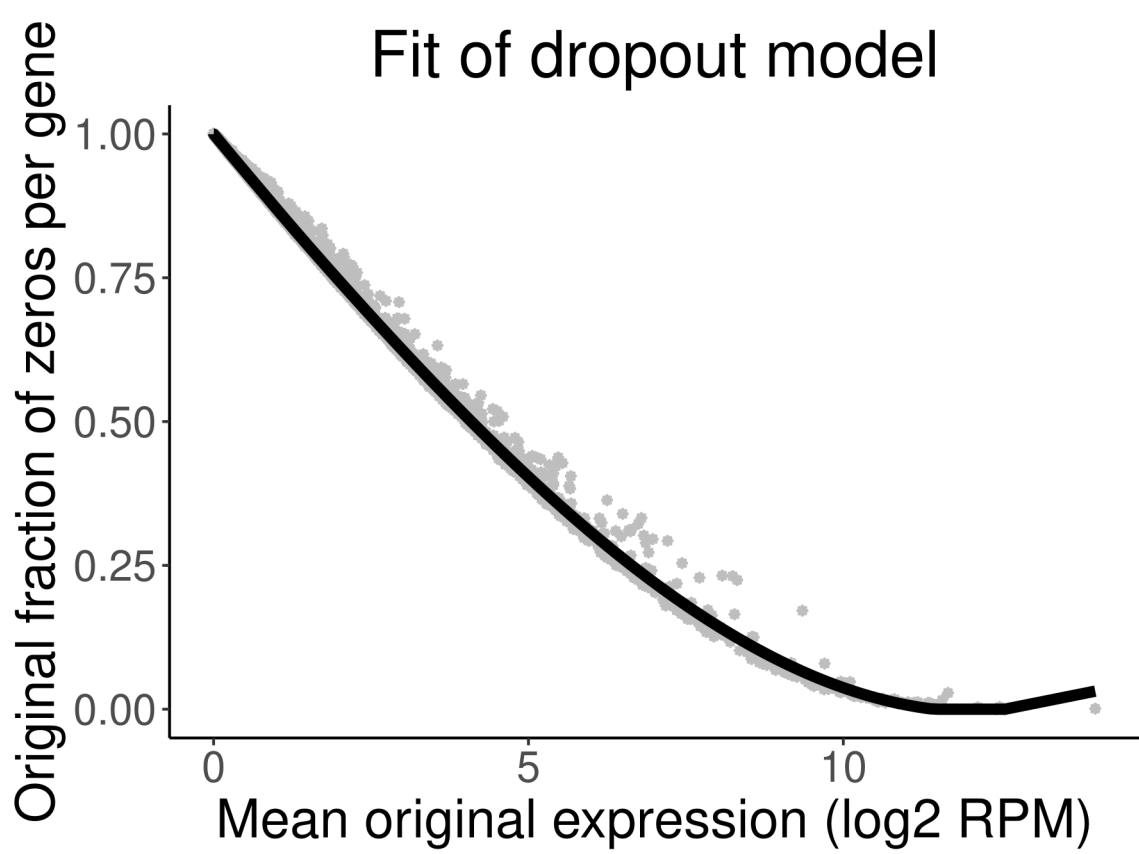

**D)**

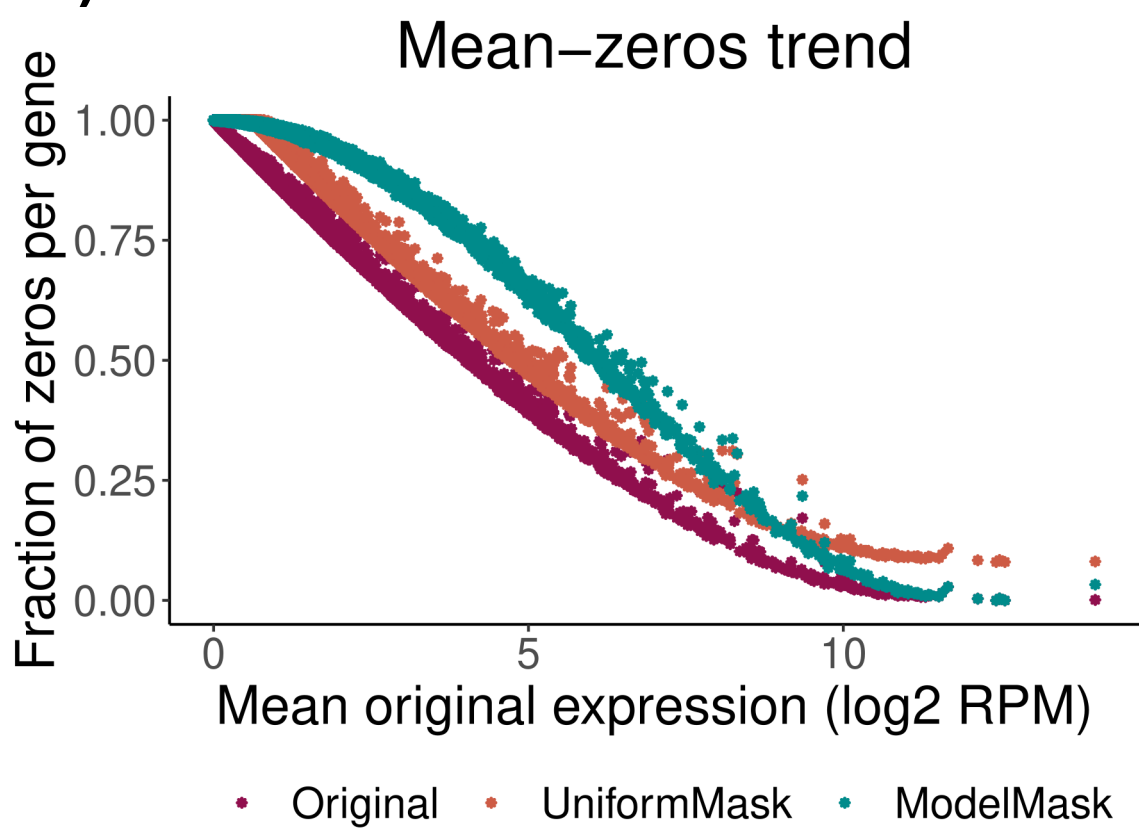

**F)**

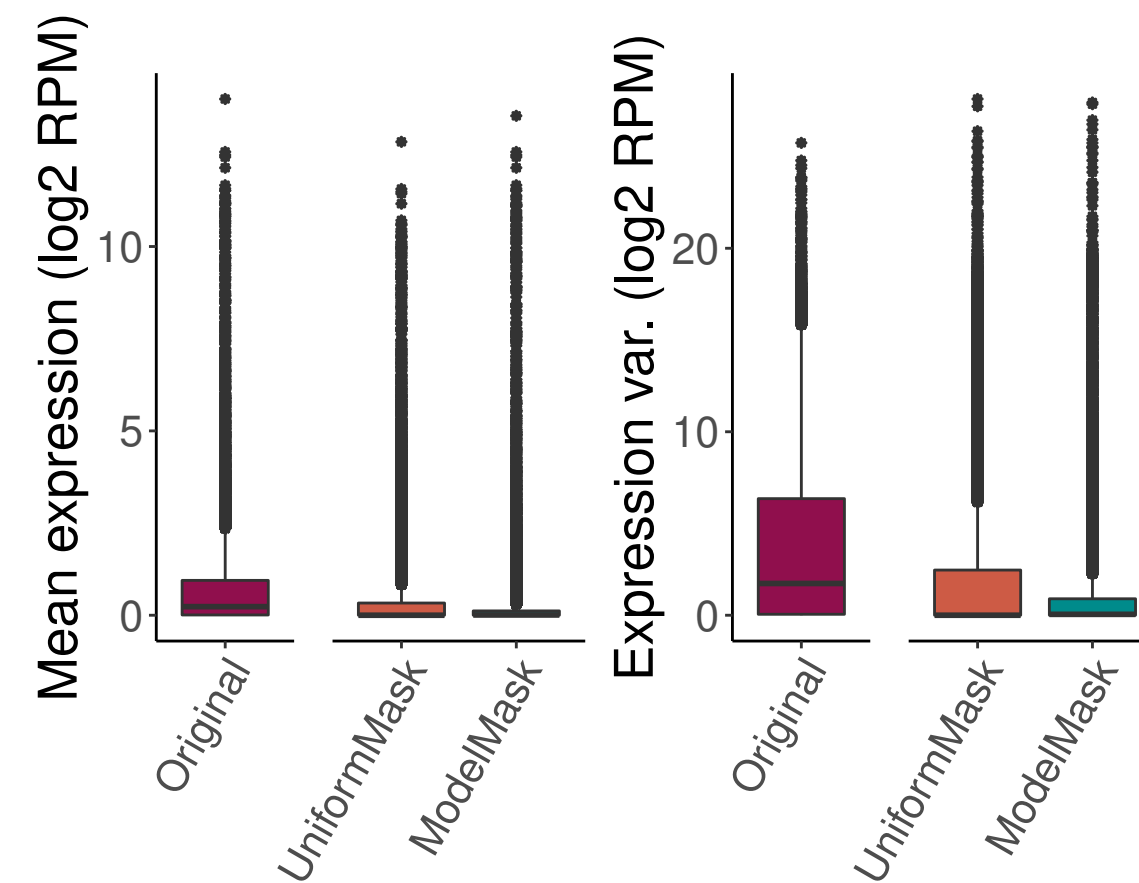

**H)**

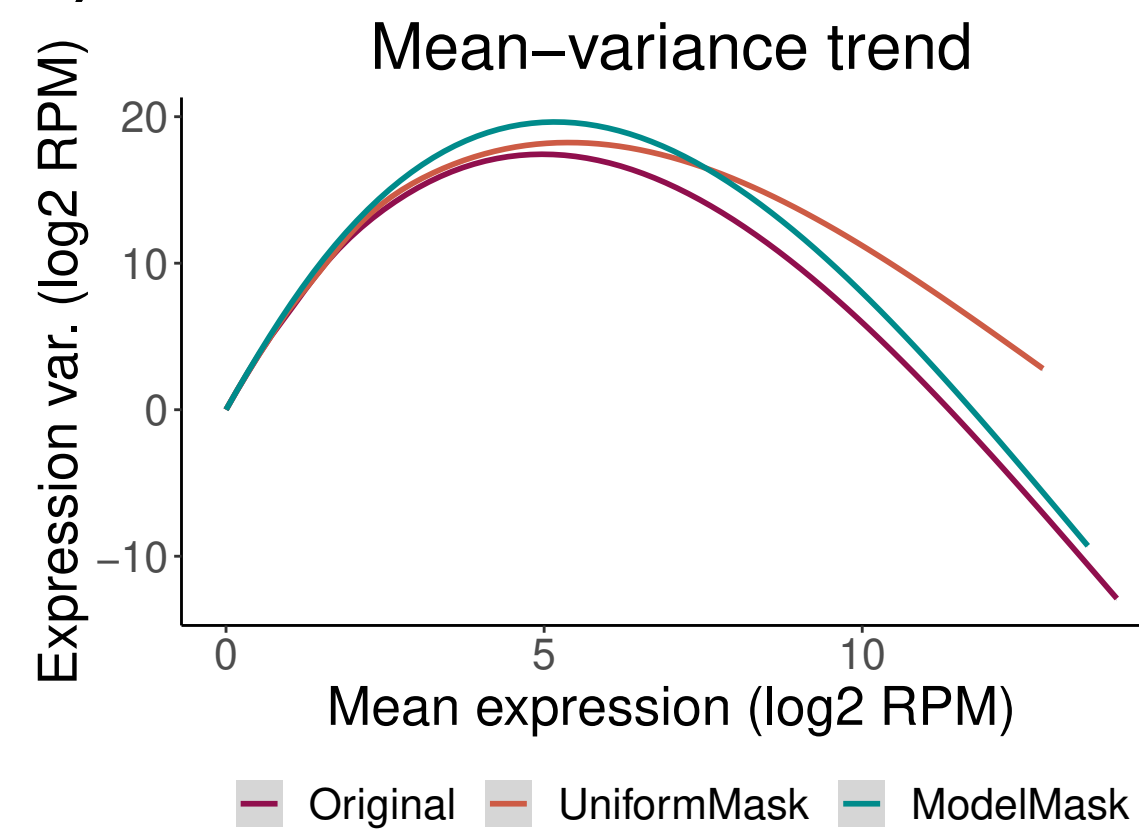

Supplement: S8 Fig — Comparisons were done on representative datasets of non-UMI data (A), C), E), G)) and UMI data (B), D), F), H)). A) and B) Fit of the spline model (Methods) to the original data. C) and D) Fraction of zeros in the data before (Original) and after (UniformMask, ModelMask) masking, compared to original average gene expression,. E) and F) Distribution of mean expression and expression variance before and after masking, compared to original average gene expression. G) and H) Mean-variance trend before and after masking. (PDF) [file pcbi.1009849.s012.pdf]

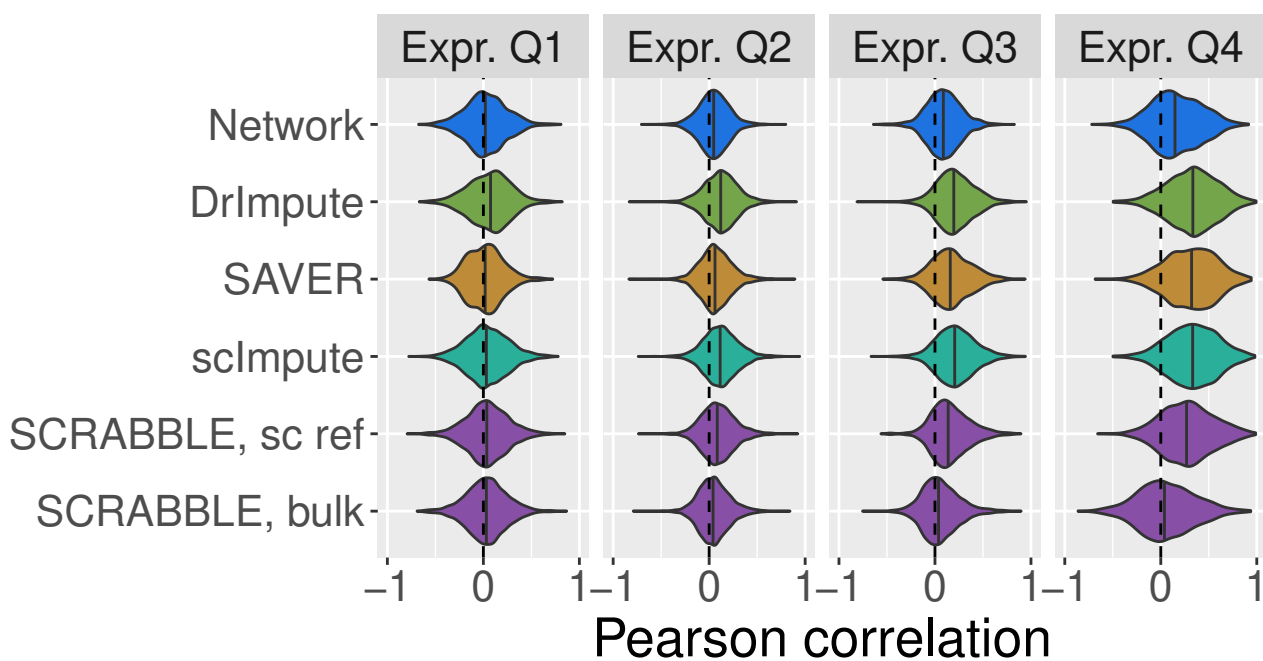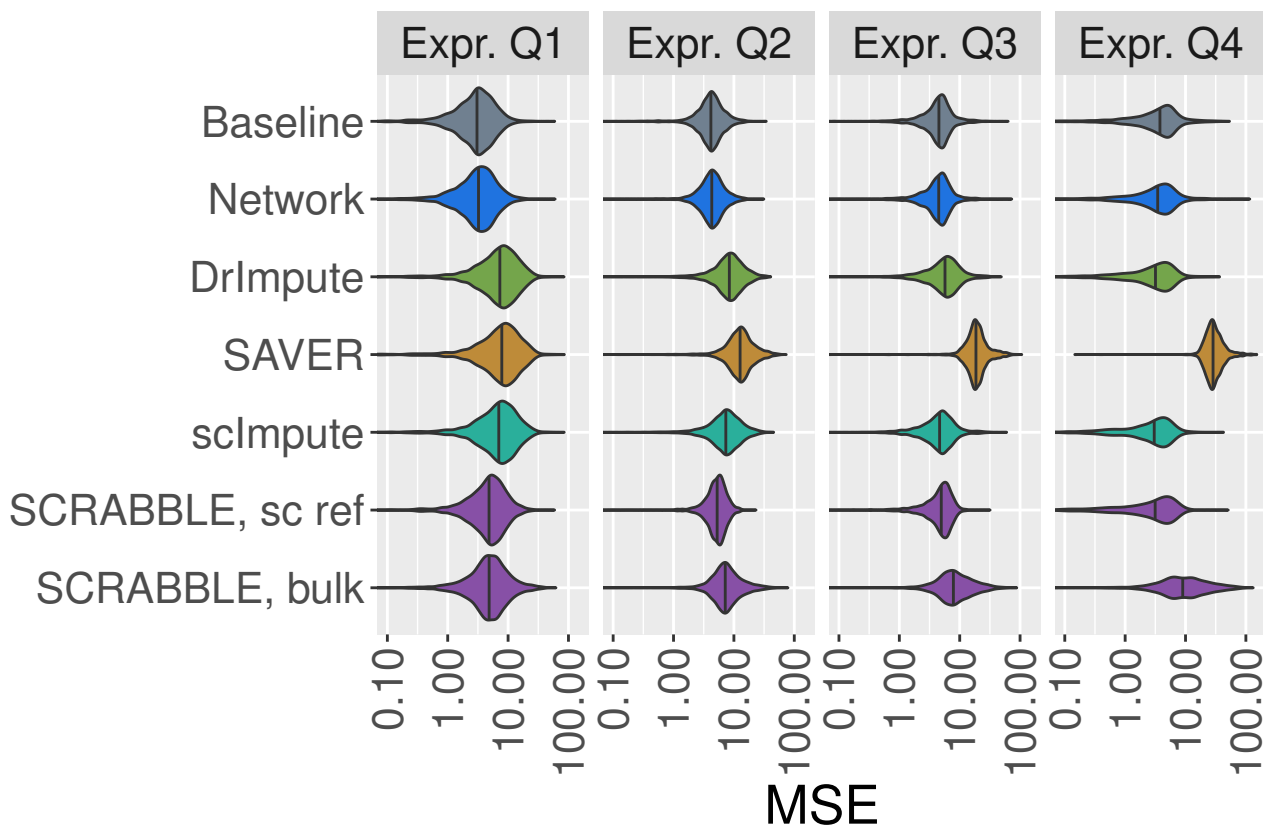

Supplement: S9 Fig — Correlation coefficient between imputed and original values (top) and MSE of imputation (bottom), upon random masking of 30% of the quantified genes in each cell in the hESC differentiation dataset. Only values that could be imputed by all methods were used for performance analysis. Expression quartiles are determined on the masked data. The MSE axis is presented log-transformed and was cropped at 0.1 to exclude the low-MSE tail from visualization and facilitate result comparison. kNN-smoothing is not included in the comparison since no imputations could be performed by this method. (PDF) [file pcbi.1009849.s013.pdf]

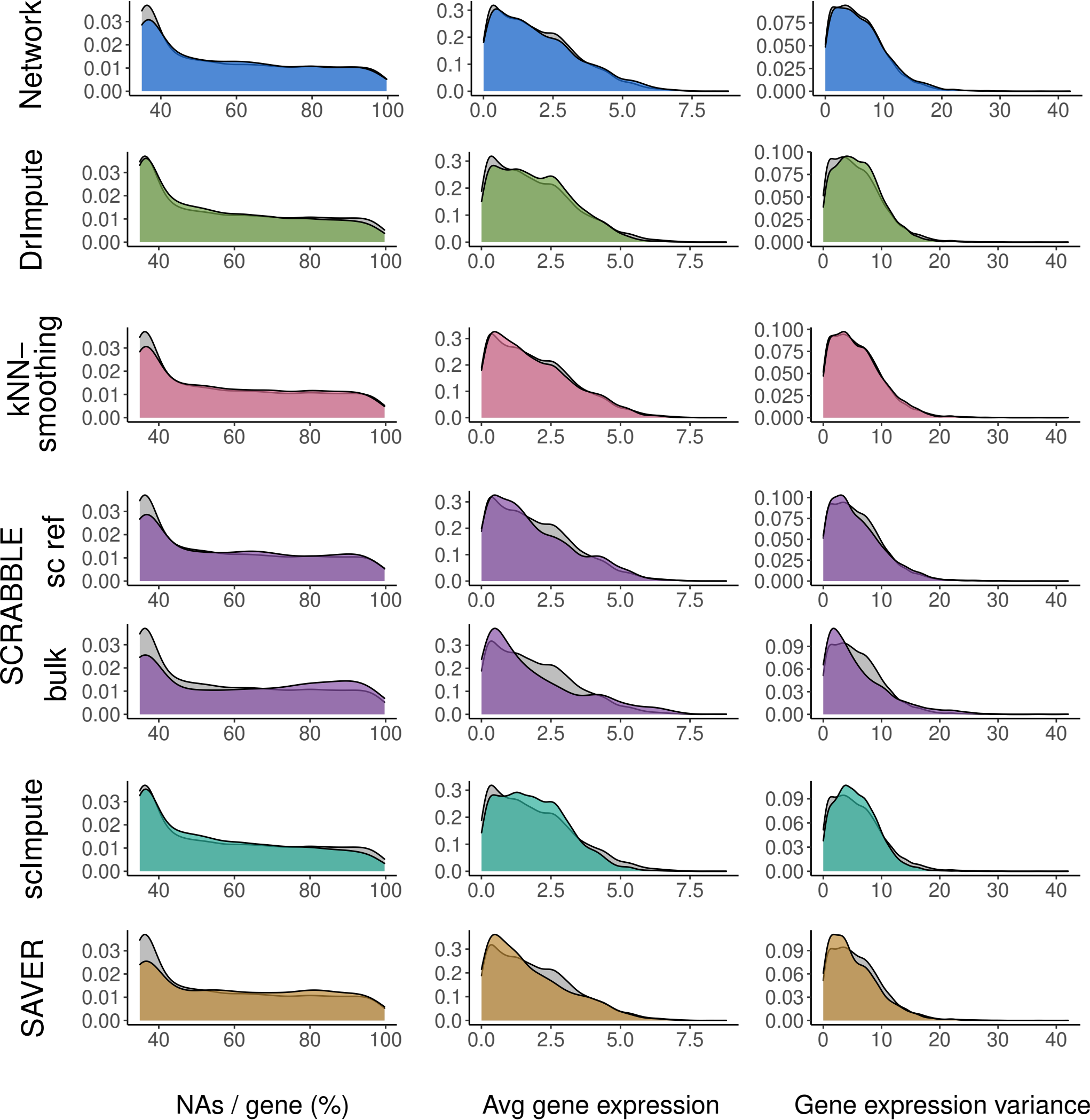

Supplement: S10 Fig — Distribution of missing values per gene, average expression levels and variance of the genes for which a given method is one of the top performers, compared against all tested genes (background). Average gene expression is shown as log2-transformed normalized expression. Methods with close performance to the best method (correlation not smaller than 0.1 –best method) are selected as top best performing. Genes for which all methods are best performers are included in the background but not in the foreground. (PDF) [file pcbi.1009849.s014.pdf]

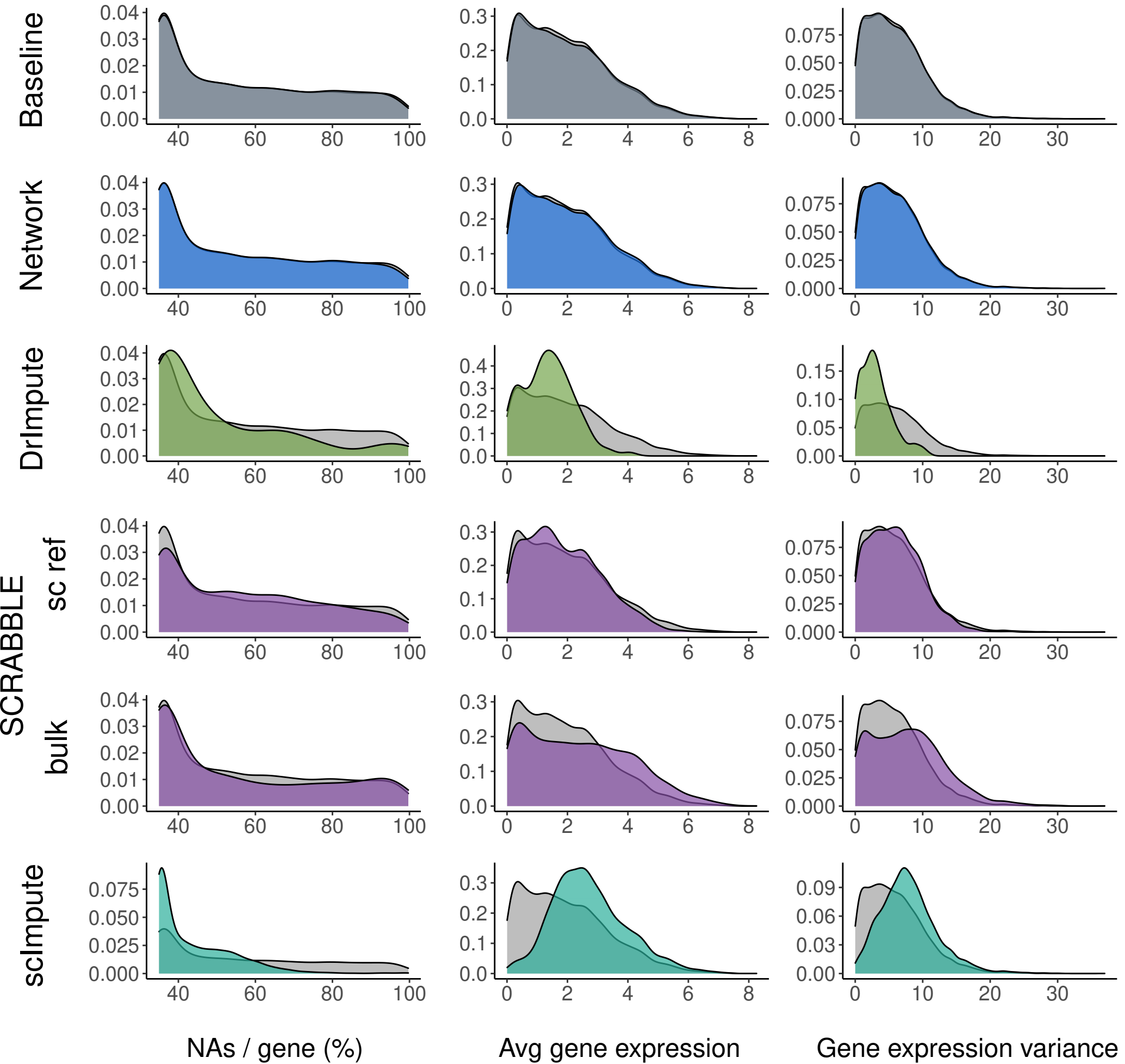

Supplement: S11 Fig — Distribution of missing values per gene, average expression levels and variance of the genes for which a given method is one of the top performers, compared against all tested genes (background). Average gene expression is shown as log2-transformed normalized expression. Methods with close performance to the best method (MSE not higher than 1/20 of the MSE range for that given gene) are selected as top best performing. Genes for which all methods are best performers are included in the background but not in the foreground. (PDF) [file pcbi.1009849.s015.pdf]

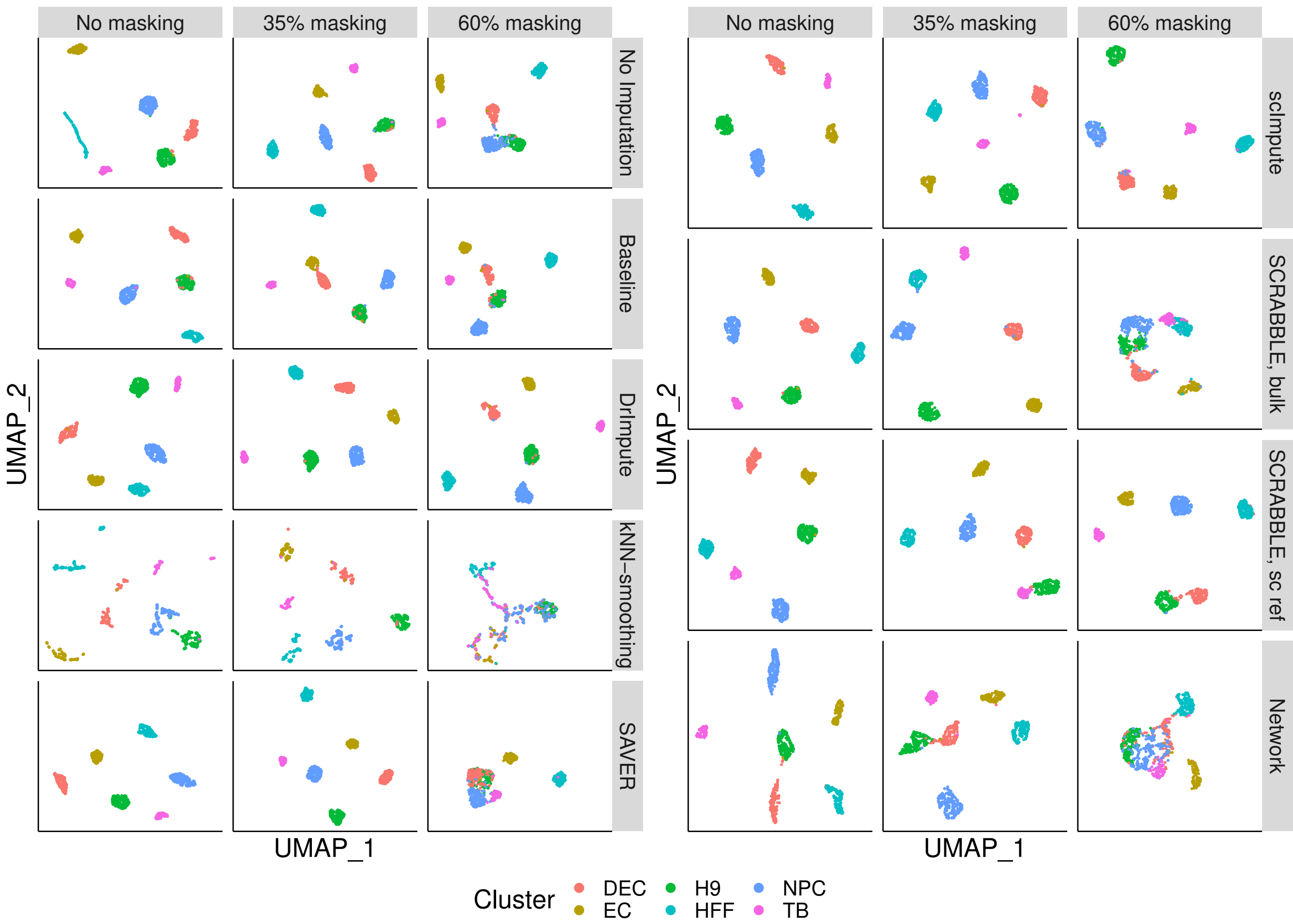

Supplement: S12 Fig — Data was subject to: no masking (left column), relaxed masking (35% of quantified entries per gene were set to zero, middle column), stringent masking (60% of quantified entries set to zero, right column). The plot in the upper left reflects the clustering on the original, unchanged data. Imputation was performed for actually missing values in the original data (all columns) and on masked values (columns 2 & 3). Colors represent cell type label annotations from the original publication. DEC: definitive endoderm cells; EC: endothelial cells; H9: undifferentiated human embryonic stem cells; HFF: human foreskin fibroblasts; NPC: neural progenitor cells; TB: trophoblast-like cells. (PDF) [file pcbi.1009849.s016.pdf]

ZINB-WaVe

K = 2

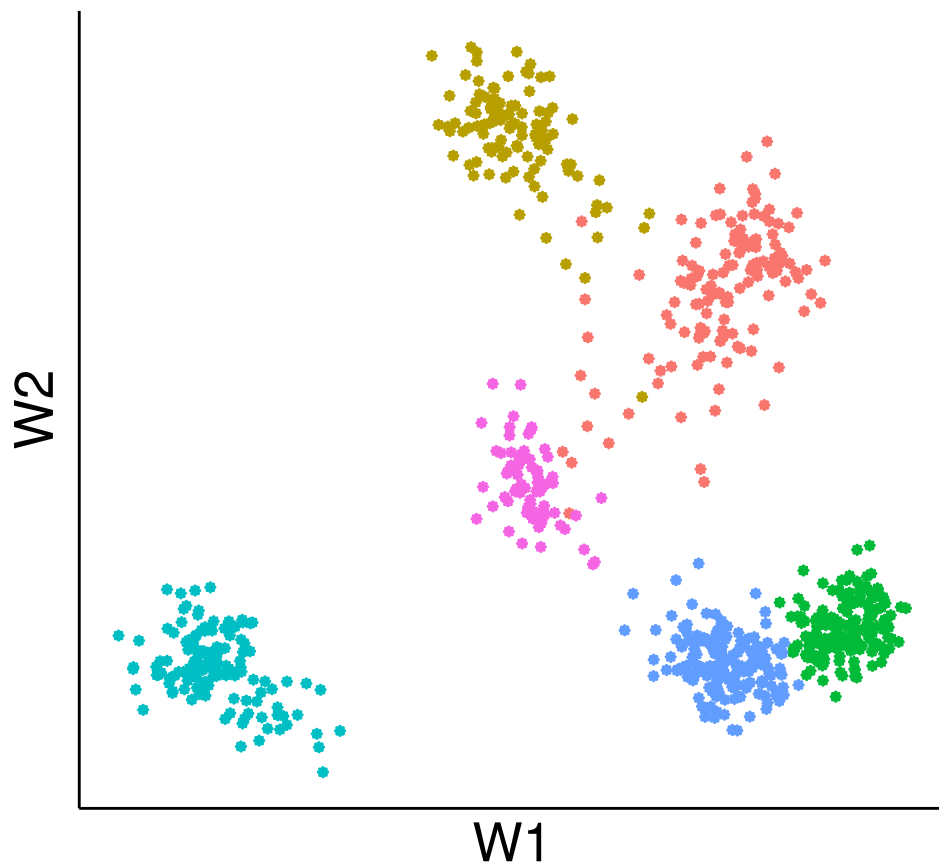

tSNE

5 PCs

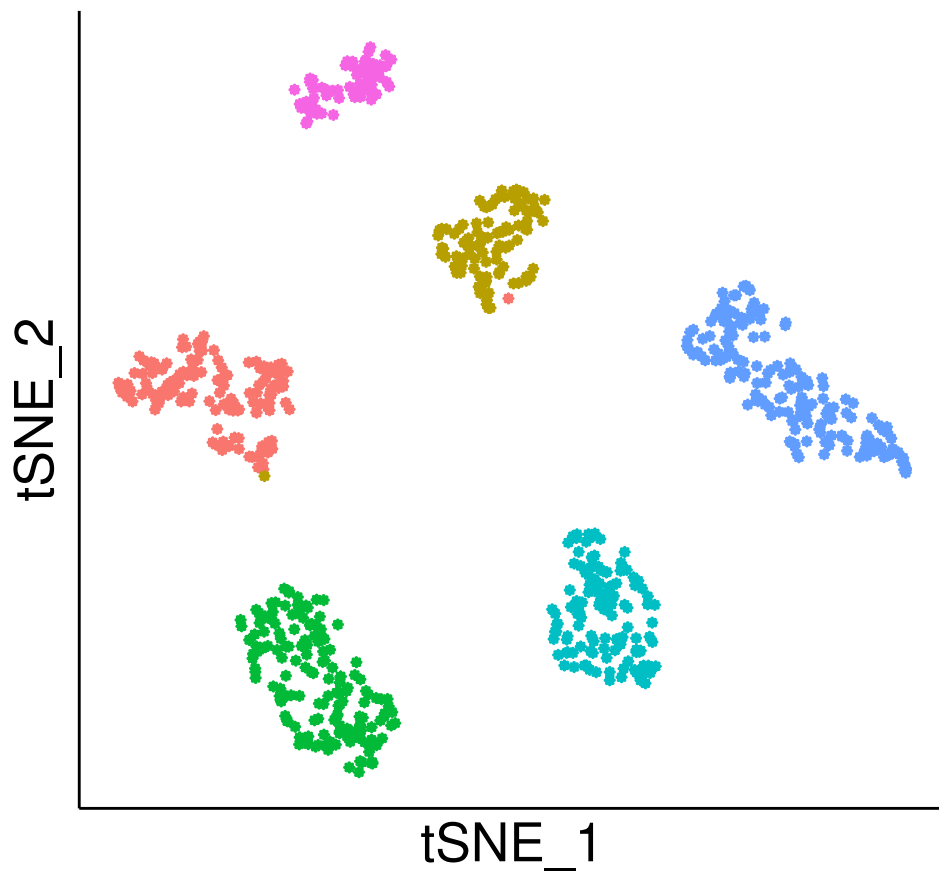

Cell type

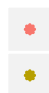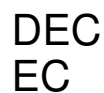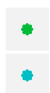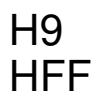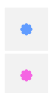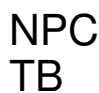

Supplement: S13 Fig — Data was not subject to any masking. Colors represent cell type label annotations from the original publication. DEC: definitive endoderm cells; EC: endothelial cells; H9: undifferentiated human embryonic stem cells; HFF: human foreskin fibroblasts; NPC: neural progenitor cells; TB: trophoblast-like cells. (PDF) [file pcbi.1009849.s017.pdf]

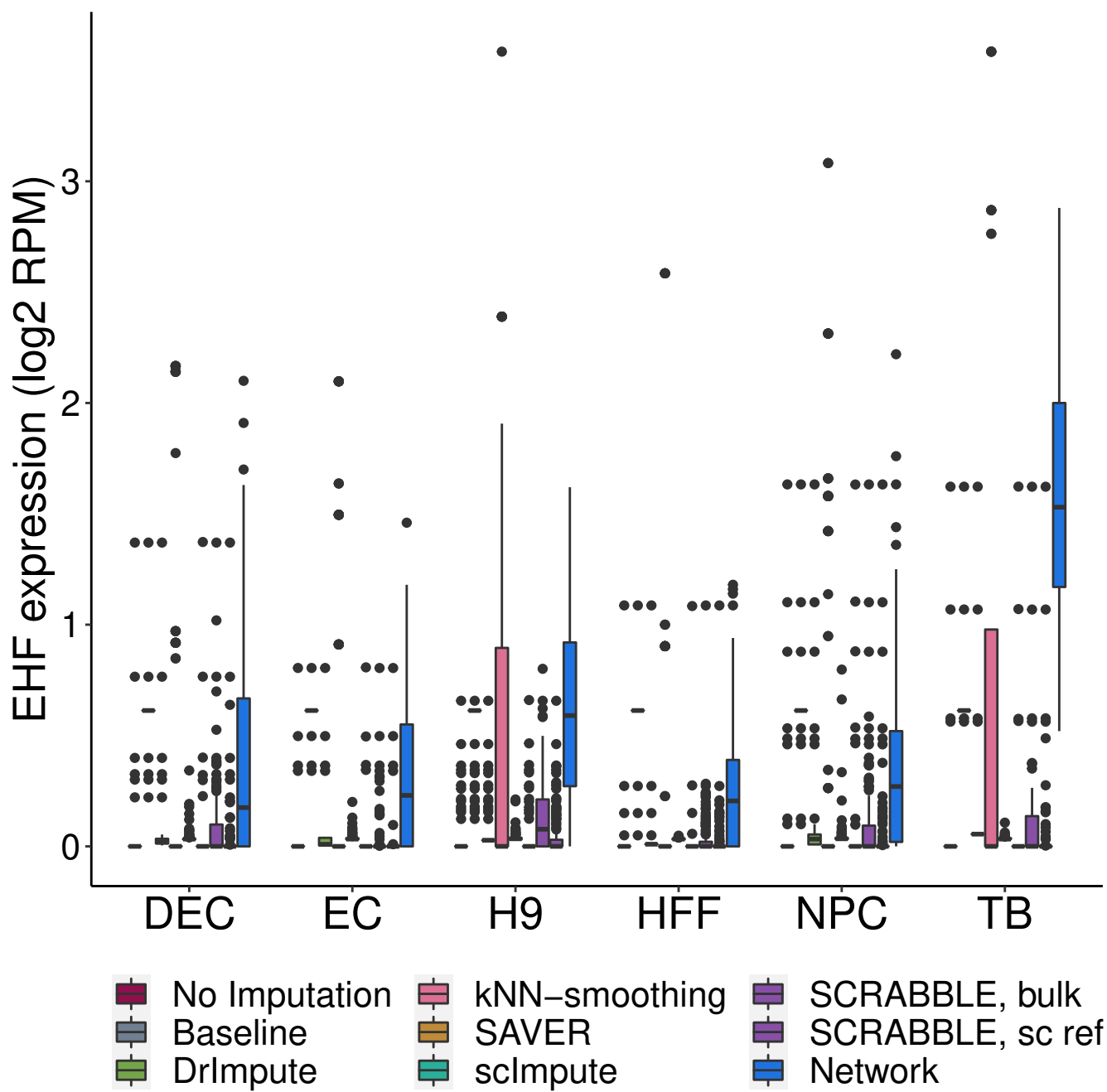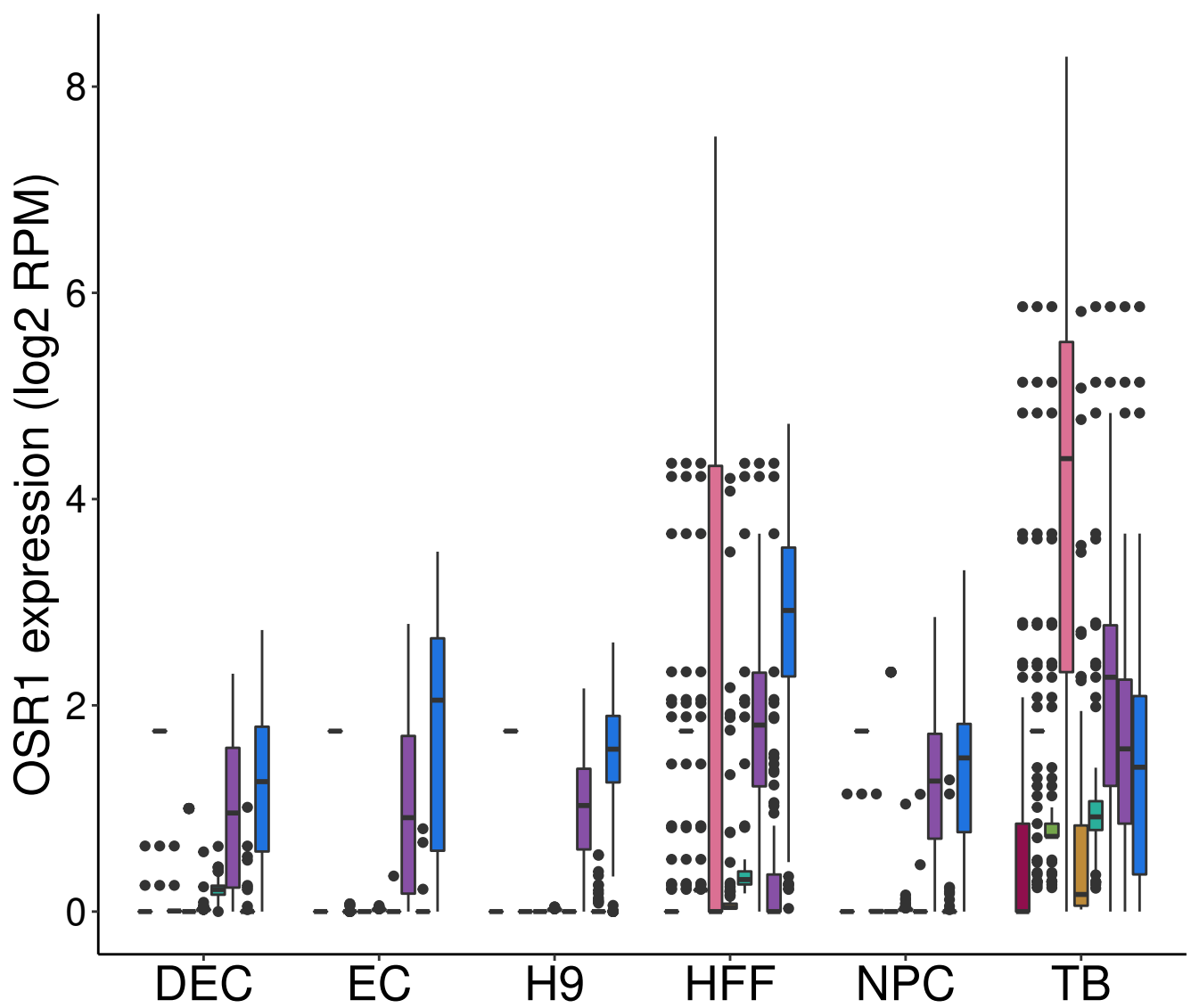

Supplement: S14 Fig — DEC: definitive endoderm cells; EC: endothelial cells; H9: undifferentiated human embryonic stem cells; HFF: human foreskin fibroblasts; NPC: neural progenitor cells; TB: trophoblast-like cells. (PDF) [file pcbi.1009849.s018.pdf]

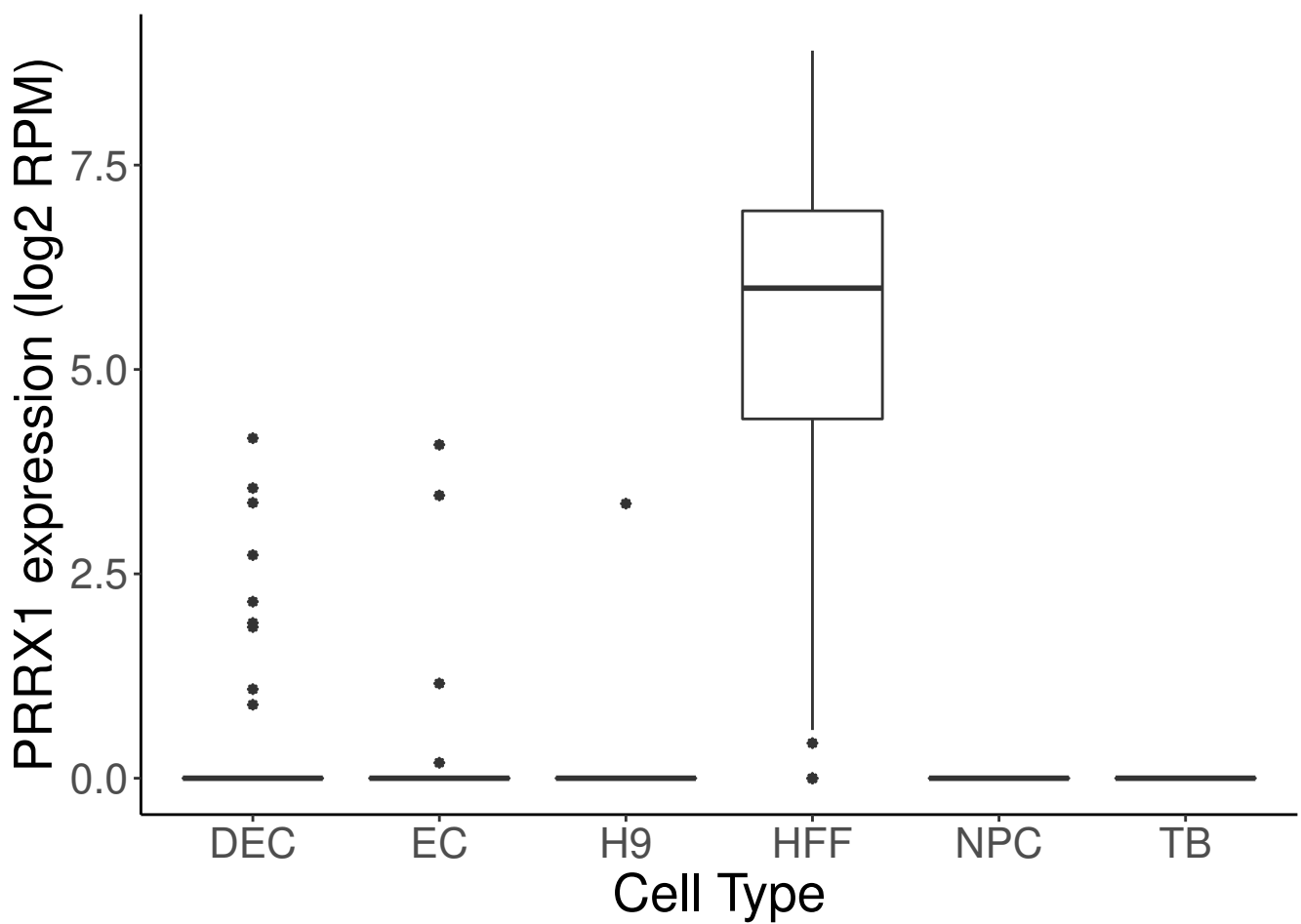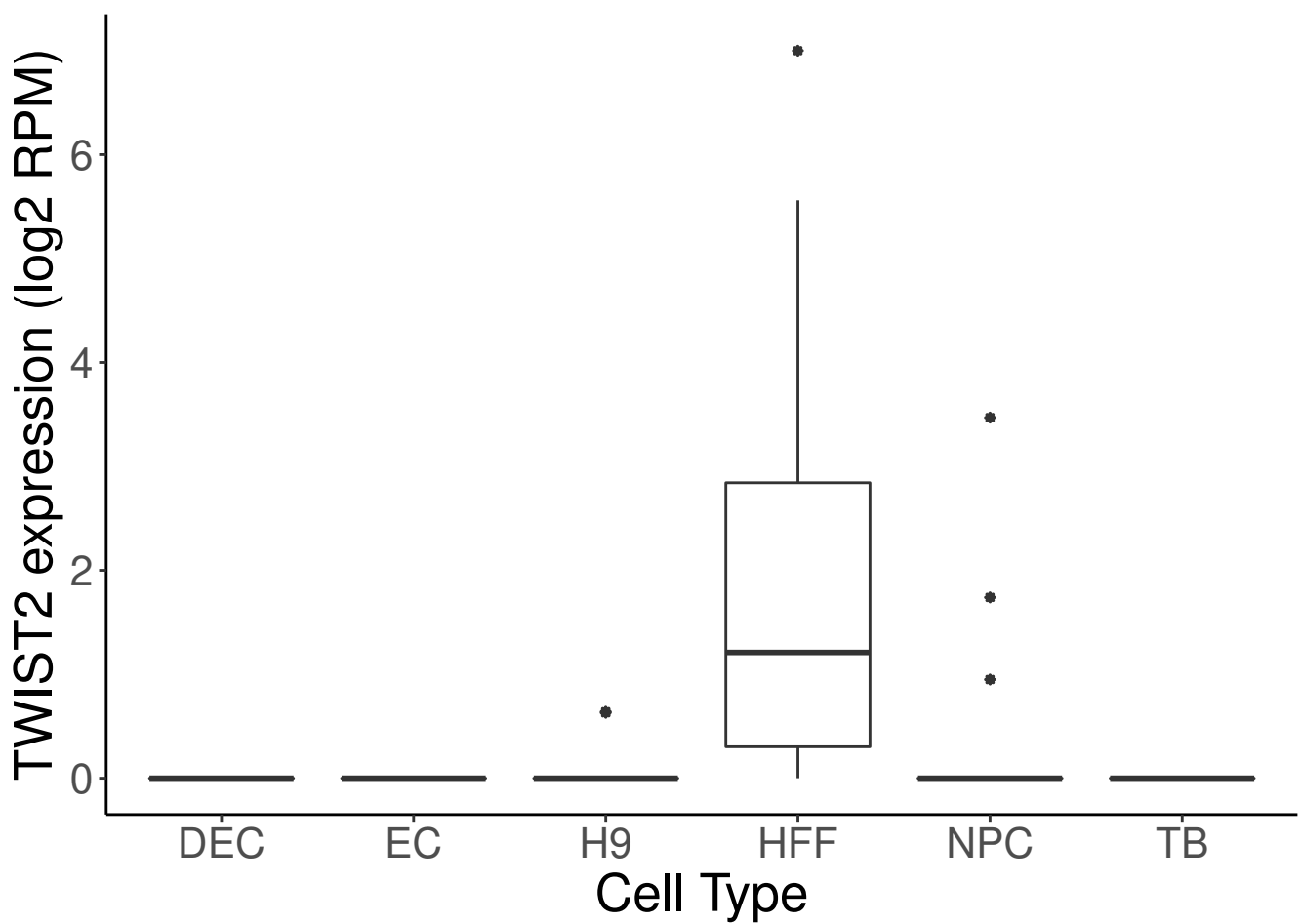

Supplement: S15 Fig — DEC: definitive endoderm cells; EC: endothelial cells; H9: undifferentiated human embryonic stem cells; HFF: human foreskin fibroblasts; NPC: neural progenitor cells; TB: trophoblast-like cells. (PDF) [file pcbi.1009849.s019.pdf]
