# Supplementary material for: Regulatory network-based imputation of dropouts in single-cell RNA sequencing data
Source: PLoS Comput Biol. 2022 Feb 17;18(2):e1009849. doi: 10.1371/journal.pcbi.1009849 (PMC8890719; doi:10.1371/journal.pcbi.1009849)
Supplement: Attachment — Submitted filename: ResponseLetter.pdf [file pcbi.1009849.s020.pdf]

Dear Dr Zou and Dr. Ioshikhes

Thank you very much for subjecting our manuscript “Regulatory network-based imputation of dropouts in single-cell RNA-sequencing data” to peer review and for inviting a revised version. We very much appreciate the constructive comments of the three reviewers, which have helped us to greatly improve our work.
In response to the reviewer comments we have (i) added kNN-smoothing as an additional imputation method, (ii) we have added pseudo-time trajectory analysis as another application/benchmarking of single cell dropout imputation, (iii) we have substantially revised Figure 3 (which is now Figure 4), and (iv) completely revised the text to improve its clarity.
We hope that these changes will convince you and the reviewers. Therefore, we are looking forward to your reply!

With best regards
Andreas Beyer

Note: Reviewer comments are shown in *italic*. Our replies are shown in green.

Reviewer #1: *In this manuscript, the authors proposed a transcriptional regulatory network learned from bulk gene information for single-cell RNA sequencing data imputation. The network approach showed good performance for lowly expressed genes, which could be utilized for the imputation of markers and regulators. Additionally, the authors make a point that there is no best imputation approach but only the best method for a particular combination for different circumstances. Overall, the manuscript is well organized. I have some suggestions for the authors to improve their work.*
We thank the reviewer for this encouraging feedback!

1. The authors argued that the “Baseline” method, which simply calculated the average expression value of the gene, performed well for the imputation. It is astonished that such a simple method achieved such good results. I believe that this simple method must have been reported and compared before in single-cell imputation field. I would suggest the authors to find related papers and add more discussion to this finding.
To our knowledge, the average has not been proposed or benchmarked as an imputation method. As we pointed out, we were surprised ourselves and Baseline only makes sense when using the MSE as a quality criterion:
“A surprising finding of our analysis is the fact that the sample-wide average expression (‘Baseline’) performs well for the imputation of many genes when using the MSE as a performance measure …”
In order to explain our initial motivation we added the following statement to the Discussion (line 466):
“Originally, we developed Baseline only as a benchmark of a minimalistic approach in order to compare the performance gain of more sophisticated approaches against this one. Much to our surprise, many genes could not be imputed better by any of the competing approaches.”
Further, in order to stress the point that Baseline is only of limited use in real-world applications, we added the following sentence:

“Obviously, Baseline does not support the identification of differentially expressed genes between different groups of cells. However, it may help reducing artifacts resulting from the clustering of cells with technical zeros (Fig. 3).”

2. Hou et al. have made a terrific benchmark work for 18 single-cell RNA sequencing imputation methods:
Hou, W., Ji, Z., Ji, H. et al. A systematic evaluation of single-cell RNA-sequencing imputation methods. Genome Biol 21, 218 (2020).
Based on their conclusion an important state-of-the-art method, kNN-smoothing, was missing in this manuscript. I suggest the authors to add this method in this work.
We thank the reviewer for bringing this method to our attention. We have added kNN-smoothing to the method comparison in both performance evaluation according to correlation and MSE, and downstream analyses. kNN-smoothing is conceptually similar to other imputation methods like scImpute and DrImpute as all of them ‘borrow’ information from neighboring (i.e. similar) cells.
In our analysis, kNN-smoothing did not outperform other methods in imputing dropouts whose real expression was known. Further, in our analysis kNN-smoothing did not seem to provide an advantage over other methods for trajectory inference (new Figure 3) and may lead to the detection of false positive cluster markers (new Figure 4, previously Figure 3). Please, note that Hou *et al*. used a different evaluation scheme. For example, they did not quantify the methods’ ability to recover masked measurements, as we did. This may partly explain the different conclusions. Further, it should be kept in mind that kNN-smoothing was developed with UMI-based data in mind, which has a different distribution than plain read counts without UMI information. The fact that our downstream analyses were done using read count data may lead to different conclusions from those presented by Hou *et al*.

3. The URL “http://www.tfcheckpoint.org/index.php/browse” in line 581 is not accessible, please check the link.
The link was updated and the corresponding reference added.

4. More details of previously learnt regulatory models was encouraged to be added.
We admit that it is not entirely clear to us, which additional details the reviewer is asking for. The method of network learning, the input data used for the learning, and the testing and evaluation of the network are described in the Results and Methods sections, while figures are shown in the supplement (see Supplementary Figures 1 & 2).
In order to address the reviewer comment we have added a discussion on potential input networks to the R package (and their advantages) to the Discussion section (line 500): “While, by default, ADImpute makes use of the transcriptional regulatory network described here, the user can provide any desired gene network e.g. for a different species. Our network has been trained on a large set of human cancer cell lines and thus is expected to capture much of the possible regulatory interactions of interest to many users; however, ADImpute facilitates the use of custom-made more specialized networks.”

5. Several abbreviations don’t have their full names for their first appearance, for instance, “MAGIC“ in line 77.
This has been changed in the manuscript for all abbreviations used in the main text.

Reviewer #2: *The paper aims to improve the performance of imputation on scRNA-Seq data through a transcriptional regulatory network learned from external, independent gene expression data. This study implemented an R-package called ADImpute that automatically determines the best imputation method for each gene in a dataset. The methods and results reported in this paper demonstrate that their network-based approach outperforms published state-of-the-art methods. The work proposed that imputation should maximally exploit external information and be adapted to gene-specific features, such as expression level and expression variation across cells.*

This manuscript is a reader-friendly and well-written manuscript. The following concerns need to be addressed.
We thank the reviewer for the positive feedback!

Major issues:
1. As far as I know, the zero values in scRNA-Seq data are partly due to dropout events and partly due to gene expression. How can the authors ensure that the method proposed to impute the dropout zeros rather than incorrectly filling gene expression?
A paragraph has been added to the Introduction, explaining the challenges that biological zeros pose to dropout imputation (lines 73-82). Identifying biological zeros and imputing missing values are actually two distinct problems. Whereas our work focusses on the latter, assessing different approaches for zero *calling* is out of scope of this submission. However, our package provides the option to identify biological zeros in the data, making use of the previously published scImpute method (Li WV, Li JJ. An accurate and robust imputation method scImpute for single-cell RNA-seq data. Nat Commun. 2018). Users are free to use other methods for calling biological zeros.

2. The challenges of improving the imputation of scRNA-Seq should be explained in-depth, such as what are the main challenges in the study of imputation methods and how these studies do may overcome these challenges.
A paragraph has been added to the Introduction, explaining the challenges that biological zeros pose to dropout imputation and the danger of introducing false positives in downstream analyses (lines 73-82). This new text plays together with the subsequent paragraphs in the Introduction that cover additional challenges of dropout imputation.

3. The proposed model was compared with several methods, but lack of discussion of details regarding the weaknesses. Could the authors give more analysis about why the proposed model does better than previous work? More insightful discussions should be given.
A discussion of the weaknesses of previously described methods is already provided in the Introduction (lines 83 to 103). We added more details in the Introduction regarding the limitations of SAVER and G2S3 (line 110): “However, if the scRNA-seq dataset at hand is sparse, the usefulness of the gene-gene relationships learnt from that dataset can be limited.” We also added a sentence regarding the limitations of SCRABBLE (line 115): “Limitations of this approach are that first, a matching bulk RNA sequencing data set needs to be available and second that the method only uses external data to adapt the distribution of the single cell data, but does not use it to inform gene-gene relationships.”
Next, we have reworked Figure 2, which evaluates the criteria under which each method has a performance benefit to explain the phenomena using empirical data.

Further, we added the following section to the Discussion (line 445): “scImpute, kNN-smoothing and DrImpute elevated the number of cluster markers found in downstream DE analysis (Figure 4), but these additional markers seemed to dilute the true biological signal in the data. A similar behaviour has been described elsewhere for scImpute (2). Similarly to our network-based approach, SAVER makes use of gene-gene relationships for imputation. However, SAVER learns these relationships in the dataset at hand, while Network makes use of externally trained relationships. The fact that Network outperforms SAVER in our comparisons suggests that the scRNA-seq data at hand may often be too sparse for relationships to be adequately learnt.”

Reviewer #3: *In this paper, Leote et al. proposed a regulatory network-based imputation method for dropouts in scRNA-seq data. I have the following concerns regarding this manuscript.*

1. For the evaluation of imputation methods, although the authors used synthetic data and simulation, their real data analysis was rather limited. The only real data results are in the section “Network-based imputation uncovers cluster markers and regulators.” However, other commonly used downstream analyses, including clustering, DE gene analysis, and cell trajectory inference, were not evaluated on the imputed data. The authors should add more real data results to demonstrate that their imputation methods can benefit downstream analyses better than existing imputation methods do.

We intentionally did not use any simulated gene expression values. Instead, our analyses make use of masking on real datasets (i.e. on top of the existing sparsity, setting a percentage of the non-zero observations per gene to zero and treating them as dropouts). This approach (i) does not change the distributions of gene expression values, (ii) maintains real gene-gene relationships without having to simulate them, and (iii) only uses real, measured gene expression values as a ground truth. Thus, all our results are based on real data.
Using that real data we evaluated the following aspects of single cell data analysis: (a) correctly estimating single expression values (Fig. 1 and Suppl Fig. 4, 5 & 6); (b) identifying cell cluster markers (Fig. 4); (c) projections and visualization of single cells (Suppl. Fig. 12 & 13), and (d) identifying transcriptional regulators (Fig. 5 and Suppl. Fig. 14).
We thank the reviewer for suggesting pseudo-time trajectory inference as another application of dropout imputation. Thus, in order to even better assess the dropout imputation methods, we have added a trajectory inference analysis on non-imputed and imputed data (new Figure 3), which is – again – based on real data only.
Further, we also re-arranged the main text in order to better highlight the DE analysis previously done.

2. If authors want to claim that their imputation method can benefit cell marker detection, the first step should be to find meaningful cell clusters and label them as cell types. However, the cell clustering results were not shown.

Since the cell clusters were already identified by the authors of the original publication, we have refrained from repeating this analysis. Instead, our approach uses those independently generated clustering results to evaluate the performance in marker detection for each method.

3. I do not find the current real data results convincing. For example, in Figure 3A, there is little difference between the cluster markers found on the imputed data (by the proposed method) and on the original data. Hence, this figure did not make a strong point.

The main message we intended to get across with Figure 3 (now Figure 4) is that some methods, like scImpute, lead to excessively large numbers of cluster markers detected. The remaining panels of the figure show that these extra markers do not lead to a stronger enrichment in "high confidence" GO terms that are also detected without imputation; instead they often reduced the original enrichment signal. This suggests that these extra markers may be false positives that dilute the true signals in the data. In fact, this observation is consistent with earlier work, which had shown that many imputation methods lead to artificial cell clusters (see references in the text).

In order to make Figure 3 (now Figure 4) more concise, we removed panel A, and altered panel B so it shows both the total number of cluster markers detected with each imputation strategy and the number of those which are only detected with the given imputation strategy.

4. In Figure 1, can the author label the sequencing technology for the hESC data as well? It seems that the proposed network imputation favors the UMI data. If this is the case, please clarify it.

This has been fixed across the manuscript. A comment has been added in line 230: "According to the correlation quality measure our network-based approach (called 'Network') mostly outperformed other methods (Figure 1), <u>especially in UMI-based datasets.</u>" (added words underlined)

5. For Figure 2, a distance metric should be used to quantify the similarities or differences between the background and the imputed data.

With Figure 2, our intention is to focus on the comparison of the foreground of the different methods, and not the comparison of the foreground and background for each method. Since the foreground represents the features of the genes best predicted by that particular method, we wish the reader to be able to compare the features of the genes best predicted by each method. The background is included only as a visual reference so that the reader knows how the foreground compares to the background distribution. We have reworked Figure 2, including a new scheme, to better make the point. For example, it should now be clear that DrImpute preferentially imputes genes with few NAs, while Network outperforms its competitors for genes with many NAs (see left most column).

6. For Figure 3, the labels for different imputation methods are not in the same order in the four panels, making it difficult for a direct comparison. Why was the original data omitted in panel B?

This has been fixed in Figure 3 (now Figure 4), which now has all labels in the same order. The original data was not omitted in panel B, but to better highlight it we have moved it to the first position from the left and added it to current panel C.

7. In line 465, why does a high MSE suggest better results?

This was a mistake and has been corrected.
