# Supplementary material for: Regulatory network-based imputation of dropouts in single-cell RNA sequencing data
Source: PLoS Comput Biol. 2022 Feb 17;18(2):e1009849. doi: 10.1371/journal.pcbi.1009849 (PMC8890719; doi:10.1371/journal.pcbi.1009849)
Supplement: Attachment — Submitted filename: ResponseLetter.pdf [file pcbi.1009849.s021.pdf]

Reviewer #1: I am satisfied with the answers the authors provided to my questions. As for the newly added part of the authors (lines 73-82), I would suggest the authors to add another citation concerning technical dropout:

Zhang, Z., Cui, F., Wang, C., Zhao, L. and Zou, Q. (2021) Goals and approaches for each processing step for single-cell RNA sequencing data. Briefings in bioinformatics, 22.

We thank the reviewer for this suggestion. This reference has been added in line 76 of the manuscript. We have further added another method reference in line 506.

Reviewer #2: The authors has answered all my questions.I have no more questions.

We thank both reviewers for their positive response!
